# HSF1Base: A Comprehensive Database of HSF1 (Heat Shock Factor 1) Target Genes

**DOI:** 10.3390/ijms20225815

**Published:** 2019-11-19

**Authors:** Dániel Kovács, Tímea Sigmond, Bernadette Hotzi, Balázs Bohár, Dávid Fazekas, Veronika Deák, Tibor Vellai, János Barna

**Affiliations:** 1 Department of Genetics, Institute of Biology, Eötvös Loránd University, Pázmány Péter stny. 1/C, H-1117 Budapest, Hungary; d.kowacs@gmail.com (D.K.); tsigmond@gmail.com (T.S.); bernadette.hotzi@ttk.elte.hu (B.H.); bbazsi41@caesar.elte.hu (B.B.); fazekasda@gmail.com (D.F.); 2 Earlham Institute, Norwich NR4 7UZ, UK; 3 Quadram Institute, Norwich NR4 7UA, UK; 4 Department of Applied Biotechnology and Food Science, Laboratory of Biochemistry and Molecular Biology, University of Technology, H-1111 Budapest, Hungary; deak@mail.bme.hu; 5 MTA-ELTE Genetics Research Group, Eötvös Loránd University, H-1117 Budapest, Hungary

**Keywords:** ageing, autophagy, cell adhesion, cell cycle, circadian rhythm, chromatin remodeling, heat shock factor 1, heat shock proteins, heat shock response, ribosome biogenesis

## Abstract

HSF1 (heat shock factor 1) is an evolutionarily conserved master transcriptional regulator of the heat shock response (HSR) in eukaryotic cells. In response to high temperatures, HSF1 upregulates genes encoding molecular chaperones, also called heat shock proteins, which assist the refolding or degradation of damaged intracellular proteins. Accumulating evidence reveals however that HSF1 participates in several other physiological and pathological processes such as differentiation, immune response, and multidrug resistance, as well as in ageing, neurodegenerative demise, and cancer. To address how HSF1 controls these processes one should systematically analyze its target genes. Here we present a novel database called HSF1Base (hsf1base.org) that contains a nearly comprehensive list of HSF1 target genes identified so far. The list was obtained by manually curating publications on individual HSF1 targets and analyzing relevant high throughput transcriptomic and chromatin immunoprecipitation data derived from the literature and the Yeastract database. To support the biological relevance of HSF1 targets identified by high throughput methods, we performed an enrichment analysis of (potential) HSF1 targets across different tissues/cell types and organisms. We found that general HSF1 functions (targets are expressed in all tissues/cell types) are mostly related to cellular proteostasis. Furthermore, HSF1 targets that are conserved across various animal taxa operate mostly in cellular stress pathways (e.g., autophagy), chromatin remodeling, ribosome biogenesis, and ageing. Together, these data highlight diverse roles for HSF1, expanding far beyond the HSR.

## 1. Introduction

Upon proteotoxic stress, such as high temperatures, elevated oxygen levels, heavy metals, toxins, and bacterial infections, a highly conserved cell protective mechanism called the heat shock response (HSR) is induced to preserve cellular proteostasis [1,2,3]. The HSR leads to a robust activation of genes encoding heat shock proteins (HSPs). HSPs function as molecular chaperones to help refold or degrade damaged proteins, thereby contributing to the protection of cells from protein-damaging factors. The master regulator of the HSR is an evolutionarily conserved heat shock transcription factor, HSF1, which becomes activated via trimerization and phosphorylation, and then translocated into the nucleus to promote the transcription of *HSP* genes [4,5]. In the proximal enhancer region of its target genes, HSF1 acts through a 5’ regulatory *cis* element called the heat shock responsive element (HSE). The HSE consists of at least three subsequent inverted repeats, TTCnnGAAnnTTC (where n denotes any arbitrary nucleotide) [6,7,8].

The idea that HSF1 upregulates *HSP* genes has been initiated from the 2000s. However, several genome-scale high throughput gene expression data have uncovered that numerous non-*HSP* genes are also under the control of HSF1 [9,10,11,12,13,14,15,16]. Accordingly, HSF1 has been implicated in several fundamental biological processes being independent of the HSR, including metabolism, gametogenesis, development, and ageing, as well as in various pathologies, especially neurodegenerative disorders and cancer [2,17,18,19,20,21,22,23]. These studies have also shown that HSF1-driven transcription strongly depends on the actual type, developmental state, metabolic condition, and phase of the affected cell. Whether HSF1 targets involved in these processes are specific to a given species or conserved throughout evolution has yet determined.

Here, we report the development of a novel database called HSF1base (hsf1base.org) containing a nearly comprehensive list of HSF1 target genes identified to date. In the HSF1base, direct HSF1 targets were identified by manually curating HSF1-related publications on single gene analyses. In addition, relevant high throughput transcriptomic and chromatin immunoprecipitation data were analyzed to support the biological relevance of predicted HSF1 targets. The format of the HSF1base is PSI-MITAB 2.8, an extended version of the PSI-MI tab-delimited format [24]. This uses standardized expression data denoted with a well-defined ID, thereby facilitating the accurate use of the database for systems biology. To illustrate the applicability of the HSF1base, we performed an enrichment meta-analysis for HSF1 target genes in the yeast *Saccharomyces cerevisiae*, nematode *Caenorhabditis elegans*, fruit fly *Drosophila melanogaster,* mouse *Mus musculus*, rat *Rattus norvegicus,* and human *Homo sapiens*. Meta-analysis of HSF1 targets showed that HSF1 regulates both general and tissue- and cell type-specific targets in mice and humans, suggesting cell-specific functions for HSF1 in biological processes such as cell adhesion, mitosis, cell cycle regulation, and ageing. We also identified a relatively large set of evolutionarily conserved HSF1 target genes. Taken together, this study reveals the specificity and conservation of HSF1 regulatory networks across tissues and species, and highlights that the role of HSF1 expands far beyond regulating the HSR.

## 2. Results

### 2.1. The HSF1base Database

The *HSF1base* database is based on 117 manually selected and curated relevant publications (see Table S1). It contains altogether 24,635 HSF1 target gene interactions (i.e., genes that are up- or down-regulated by HSF1 or the regulatory region of which is able to bind HSF1) derived from several model systems such *S. cerevisiae*, *C. elegans*, *D. melanogaster, M. musculus, R. norvegicus,* and human cell lines (Table 1). Note that a given interaction could be obtained with multiple times, i.e., from more than one species. Out of these interactions, 18,356 were considered as direct ones, based on evidence for physical interaction between HSF1 and the corresponding gene. In most cases, data were resulted from genome-scale ChIP-on-chip or ChIP-Seq analyses, where up- or down-regulation of HSF1 targets was not studied just the fact of HSF1-DNA binding.

Only a relatively small number (921) of direct interactions was further analyzed to provide information about the nature of HSF1 target gene regulatory interactions (positive or negative). HSF1 target genes were directly activated in 577 records while repressed in 344 cases by the protein. In 6279 records, there were no evidence for a direct interaction between HSF1 and its target gene. In these cases, HSF1-dependent regulation of target genes was demonstrated by RNA-seq or microarray studies. Altogether, the HSF1base contains 15,641 unique HSF1 target gene interactions, 11,110 of which were considered as direct targets based on evidence for physical interaction between HSF1 and the gene such as ChIP-on-chip or ChIP-Seq analyses (Table 1). HSF1-dependent gene expression was detected in 1321 direct targets (774 are activated and 547 are inhibited).

### 2.2. Strategy for Testing the Applicability of the HSF1Base

**Figure 1:**
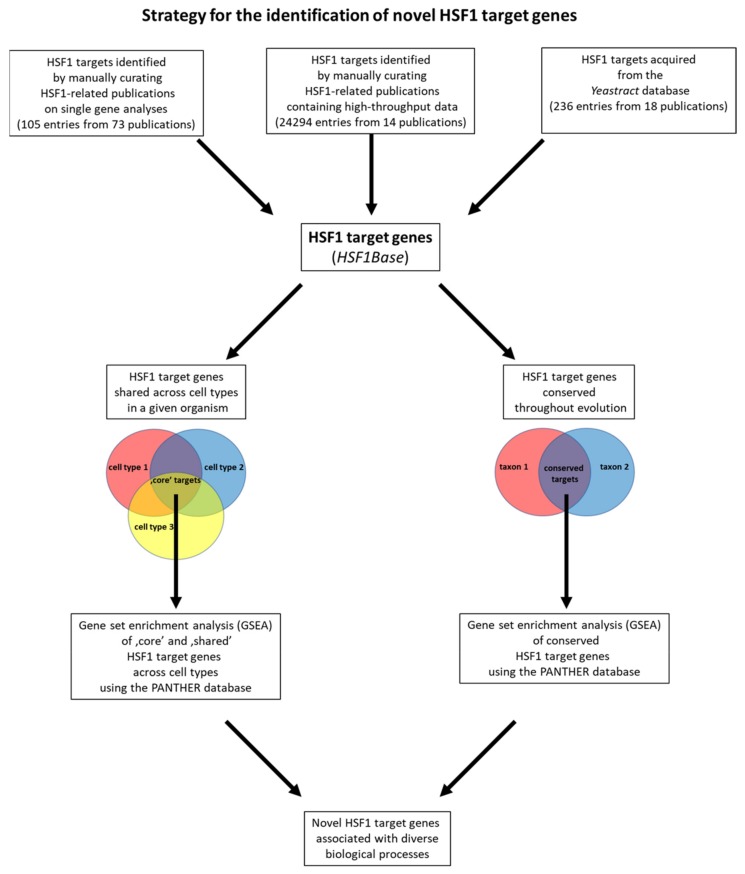
A flowchart depicting our strategy for the identification of novel potential HSF1 target genes. The database (HSF1Base) was built by manually curating HSF1-related publications on single gene analyses (105 entries from 73 publications), HSF1-related publications containing high-throughput data (24,294 entries from 14 publications) and acquiring HSF1 targets from Yeastract database (236 entries from 18 publications). Out of all target genes contained in our database we selected those shared across cell types in each organism. Then, we performed gene set enrichment analysis (GSEA) using PANTHER database on the ‘core’ (shared between all three cell types) and ‘shared’ (shared between at least two cell types) gene sets. We also selected evolutionally conserved target genes and performed GSEA using the PANTHER database on these target genes as well. We then identified novel HSF1 target genes associated with diverse biological processes of interest.

To select potential targets of HSF1 from the primary list containing every hits of high throughput analyses (Table S2), we applied the approach of Webb and colleagues (2016) who analyzed tissue specificity and conservation of FoxO (Forkhead box-O transcription factor, the effector of the insulin/IGF1 (insulin-like growth factor-1) signalling system) target genes identified by ChIP-seq binding data from various mouse cell types and in several model systems [25] (Figure 1). We determined HSF1 targets that had been described parallel in more than one cell types within a single species, and found to be evolutionarily conserved (Tables S3–S5). The resulting ‘shared’ gene sets were further applied to statistical overrepresentation tests, using the Protein Analysis Through Evolutionary Relationships (PANTHER) database [26]. In this way, gene groups were identified which are enriched within a given gene set, and can be linked to a given biological process, molecular function, protein class, PANTHER pathway or Reactome pathway. Within the gene groups we determined, individual genes were further labelled that had not previously been characterized as a HSF1 target in a single-gene-analysis study (Table S6). In addition, individual genes that had been identified to be directly regulated by HSF1 were also selected (Table S6). This approach allowed us to examine general and cell/tissue type-specific functions of HSF1, as well as its conserved and species-specific activities (Figure 1).

### 2.3. General and Tissue-Specific HSF1 Target Genes in Human

**Figure 2:**
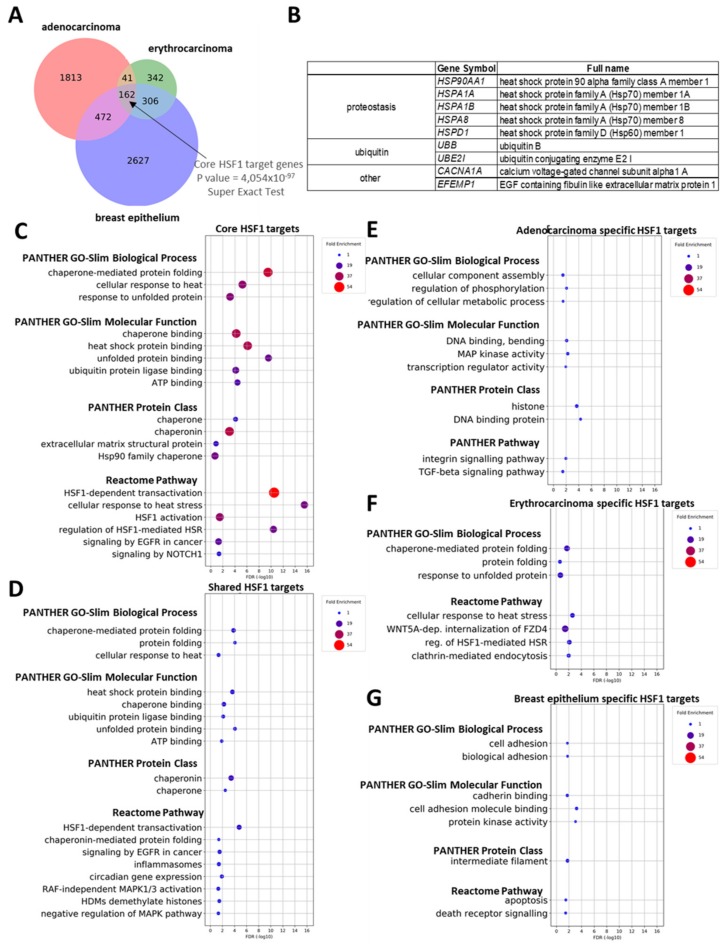
HSF1 transcription factor binds tissue-specific and shared targets in human. (**A**) Venn diagram depicting overlaps between HSF1 target genes in all cell types analysed (*p* = 4.05 × 10^−97^, super exact test; 19,608; all human genes were used as a background); (**B**) HSF1 target genes involved in ageing that are shared between all three cell types (GenAge database); (**C**) PANTHER GO-Slim Biological Process, PANTHER GO-Slim Molecular Function, PANTHER Protein Class and Reactome Pathway analysis of the 162 HSF1 target genes shared among all three cell types; (**D**) PANTHER GO-Slim Biological Process, PANTHER GO-Slim Molecular Function, PANTHER Protein Class and Reactome Pathway analysis of the 981 HSF1 target genes shared between at least two cell types; (**E**) PANTHER GO-Slim Biological Process, PANTHER GO-Slim Molecular Function, PANTHER Protein Class and PANTHER Pathway analysis of the 1813 adenocarcinoma specific HSF1 target genes; (**F**) PANTHER GO-Slim Biological Process and Reactome Pathway analysis of the 342, erythrocarcinoma-specific HSF1 target genes; (**G**) PANTHER GO-Slim Biological Process, PANTHER GO-Slim Molecular Function, PANTHER Protein Class and Reactome Pathway analysis of the 2627, breast epithelium specific HSF1 target genes. Filled circles depict statistically significant enrichments; X axis shows -LOG10 (*p*-value) *p* < 0.05; Fisher’s exact test with Benjamini–Hochberg false discovery rate correction (FDR).

To investigate which HSF1 targets are regulated in a cell type/tissue-specific manner, we compared HSF1 targets obtained from several murine and human cell lines. We observed that while some HSF1 targets are ubiquitously expressed in all human tissues examined, most of them are active in (a) specific cell types(s) only (Figure 2). Comparing the expression of HSF1 targets in each human cell type confirmed that many of these genes are active in specific cell types; e.g., 2488 genes in cervical adenocarcinoma cell lines (HeLa and HF73 cells), 851 genes in erythroleukemia-derived cell lines (K562 cells), and 3567 genes in breast epithelium (HME1, BPE, and MCF7 cell lines) (Figure 2A and Table S3). On the other hand, the expression of numerous other HSF1 target genes, specifically 17.03% of all targets (981 genes), was found in at least two cell types. These genes represent the so-called ‘shared’ HSF1 targets (Figure 2A and Table S3). Interestingly, a significant number of HSF1 target genes (162, 2.81%) are active in all cell types examined so far, representing the ‘core’ HSF1 direct targets (Figure 2A and Table S3). The list of ‘core’ HSF1 targets includes several previously identified target genes of HSF1 (e.g., *HYPK* [27], *STIP1* [28], *BAG3* [28], *JUN* [29], and *UBB* [30]), as well as targets that have not yet been characterized in detail (e.g., *CELSR1*, *JMJD6,* and *TBL1X*).

We then tested whether cell type/tissue-specific and ‘shared’ HSF1 targets have different functions, using the Protein Analysis Through Evolutionary Relationships database (PANTHER) [26]. Gene set enrichment analysis (GSEA) of ‘core’ HSF1 targets showed that most of the significantly enriched gene ontology (GO) terms are related to biological processes previously associated with a HSF1 function such as *chaperone-mediated protein folding* (e.g., *HSPA1A*, *HSPA8*, *HSPA6*, *DNAJB1*, *ST13,* and *FKBP4*) and *ubiquitin protein ligase binding* (e.g., *UBB,* and *UBC*) (Figure 2C, Tables S6 and S7). In good accordance with this observation, the most enriched molecular function terms are *chaperone binding* and *unfolded protein binding* (Figure 2C). The significantly enriched protein class terms in the ‘core’ HSF1 target gene set indeed were *chaperones* and *chaperonins* (Figure 2C). Panther pathway analysis of the ‘core’ gene set showed that the apoptotic pathway, a system known to be regulated by HSF1 [17,31,32,33], was also enriched for these genes (Figure 2C, Table 2, Tables S6 and S7). Similarly, ‘shared’ target genes were enriched for chaperone-related GO terms (Figure 2D, Tables S6 and S7). Taken together, the HSF1 ‘core’ and ‘shared’ target gene sets in three different human cell types contain genes associated with the role HSF1 in maintaining proteostasis. We also found over-represented annotations that cannot be linked to classical functions of HSF1 (Figure 2C, Tables S6 and S7). A representative example is the *extracellular matrix structural protein* term containing *LTBP4*, *CRELD1,* and *EFEMP1* genes. These are potential direct targets of HSF1, which have not been characterized in a single-gene-analysis study, and according to the HSF1base, these genes are directly controlled by HSF1. Similarly, novel HSF1 direct targets were found in the term *Signalling by NOTCH1* (*NOTCH1*, *TBL1X,* and *DTX2*). Analyzing ‘shared’ HSF1 targets revealed over-represented Reactome pathway terms such as *inflammasomes* (*BCL2L1*, *SUGT1*, *APP*, *MEFV*, *HSP90AB1,* and *NFKB2*), *circadian rhythm* (*RXRA*, *NOCT*, *NCOA2*, *BHLHE40*, *ARNTL*, *SERPINE1*, *DBP,* and *TBL1X*), *RAF-independent MAPK1/3 activation* (*IL6R*, *DUSP2*, *DUSP10*, *DUSP5*, *MAP2K2,* and *DUSP1*), *HDMs demethylate histones (PHF8*, *HIST1H4A*, *ARID5B*, *KDM6A*, *KDM4B*, *JMJD6,* and *KDM1A)* and *negative regulation of MAPK signalling* (*UBB*, *DUSP2*, *UBC*, *DUSP10*, *KSR1, DUSP5*, *MAP2K2,* and *DUSP1*) (Figure 2D, Table 2, Table S6 and Table S7). Although most of these genes have not been described as direct HSF1 targets by single-gene-analysis studies, the HSF1base identifies them as genetic factors that are directly regulated by HSF1 (for details, see Table S6). This implies that in these three cell types HSF1 exerts specific functions being independent of the HSR.

Results obtained by the PANTHER analysis of cell type-specific HSF1 target genes were different from the ‘shared’ and ‘core’ target genes. For example, target genes specific to cervical adenocarcinoma cells were most enriched for PANTHER molecular function term *DNA binding, bending* (e.g., *HIST2H2BE*, *NCAPD3,* and *NUSAP1*). Consistently, this gene set was enriched for genes associated with the PANTHER protein class term *histone* (*HIST2H2AA3, H1FX,* and *HIST2H2BE*) and genes associated with Reactome pathways terms such as the TGF-beta signalling pathway (e.g., *SMAD3*, *BMP4*, *TGFBR1,* and *SMAD7*) and integrin signalling pathway (*COL4A6*, *ITGAM*, *ITGB4*, *FN1* and *MAPK8*). In these cases, the majority of term-linked genes have not been identified as a HSF1 target in single-gene-analysis studies (Figure 2E, Tables S6 and S7).

Contrary to written above, a PANTHER analysis on HSF1 target genes in erythrocarcinoma cells did not reveal cell-specific enrichment of PANTHER terms associated with a function of HSF1 beyond maintaining proteostasis (Figure 2F, Tables S6 and S7). However, overrepresentation of Reactome-pathway-associated genes was observed, including WNT5A-dependent internalization of FZD4 (e.g., *AP2A1*, *FZD4*, *PRKCB,* and *CLTC*), clathrin-mediated endocytosis (e.g., *BIN1* and *EPS15*) (Figure 2F, Tables S6 and S7). Breast epithelium-specific target genes statistically over-represented for the term of cell adhesion biological process (e.g., cadherin coding genes (*CDH13*, *CDH12,* and *CDH3*), *Tensin-1* (*TNS1*), *Nectin-4* (*NECTIN4*), and *Teneurin-2* (*TENM2*)), and cadherin binding molecular function (e.g., *CTNND1*, *CDH4*, *PLEC,* and *CTNND2*). In this gene set, the most enriched protein class term was intermediate filament (e.g., catenin delta-encoding genes (*CTNND1* and *CTNND2*) and plakophilin-encoding genes (*PKP1* and *PKP2*)) (Figure 2G, Tables S6 and S7). In sum, these data suggest a novel function for HSF1 in cell adhesion specific to breast epithelium.

### 2.4. Cell Type-Specific and General Ageing-Related HSF1 Target Genes in Human

Since HSF1 is known to regulate the ageing process [17,18], we tested whether its target genes in any of the three cell types (adenocarcinoma, erythrocarcinoma, and breast epithelium) are associated with longevity. By overlapping the human HSF1 targets with ageing-related genes extracted from the GenAge database [34], we revealed that 117 HSF1 targets (2.08% of all genes) were previously implicated in ageing (Figure 2B and Table S8). These targets include genetic factors for insulin/IGF1 signalling (e.g., *IGF1R*, *IGFBP2*, *IRS2*, *INSR*, *FOXO1,* and *GSK3B*), stress response pathways (e.g., *SOD1*, *PRDX1*, *HSPA1A*, *HSPA8*, *HSPA1B,* and *HSPA9*), and DNA damage repair pathways (e.g., *PRKDC*, *MLH1*, *POLA1*, *RAD52*, *ERCC1,* and *RECQL4*). Out of the 10 ageing-related ‘core’ HSF1 target genes, five codes for heat shock proteins (*HSP90AA1*, *HSPA8*, *HSPD1*, *HSPA1B,* and *HSPA1A*) determine components of the ubiquitin-proteasome system (*UBE2I* and *UBB*), while the others encode the EGF- (epidermal growth factor) containing fibulin-like extracellular matrix protein 1 (*EFEMP1*), calcium voltage-gated channel subunit alpha1 A (*CACNA1A*), and a Jun proto-oncogene, the AP-1 transcription factor subunit (*JUN*). Taken together, these results convincingly show that many human HSF1 target genes are related to the ageing process.

### 2.5. ‘Shared’ and Cell Type-Specific HSF1 Target Genes in Mice

**Figure 3:**
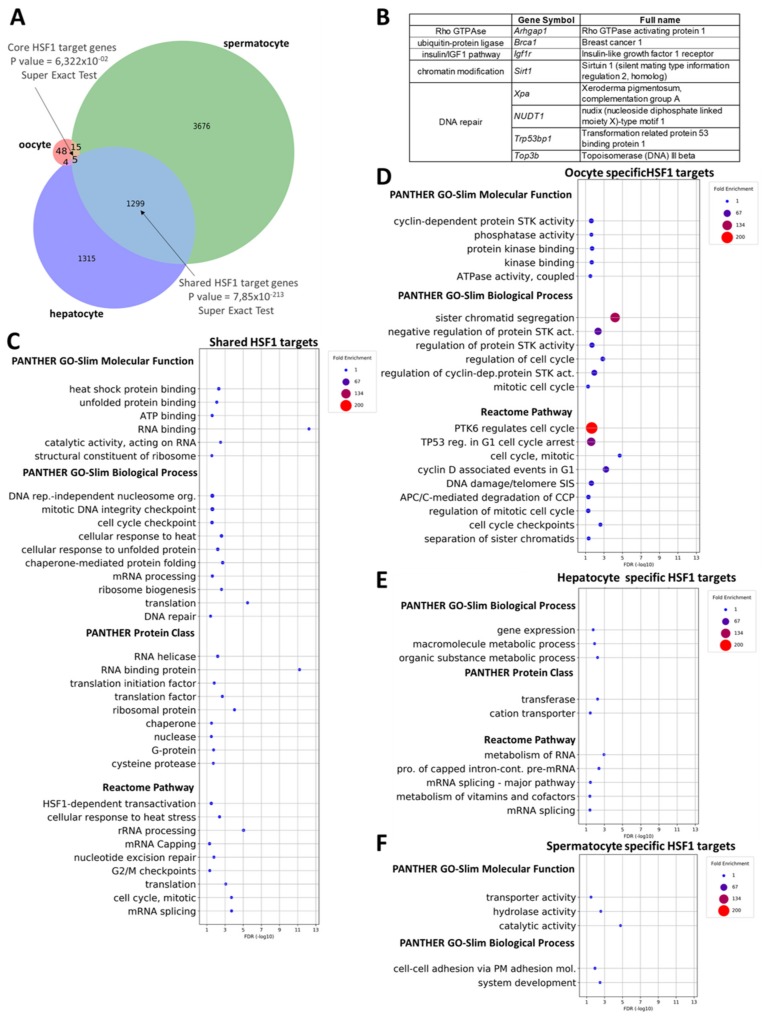
HSF1 binds tissue-specific and shared targets in mouse. (**A**) Venn-diagram representing overlaps between HSF1 target genes in all cell types analyzed. Overlaps between two datasets (blue and grey) are statistically significant (*p* = 6.322 × 10^−2^ and *p* = 7.85 × 10^−213^, super exact test; 20,993 unique mouse genes were used as a background); (**B**) HSF1 targets involved in ageing are shared between the three cell types; (**C**) PANTHER GO-Slim Molecular Function, PANTHER GO-Slim Biological Process, PANTHER Protein Class and Reactome Pathway analysis of the 1318 HSF1 target genes shared between at least two cell types; (**D**) PANTHER GO-Slim Molecular Function, PANTHER GO-Slim Biological Process and Reactome Pathway analysis of the 48, oocyte-specific HSF1 target genes; (**E**) PANTHER GO-Slim Biological Process, PANTHER Protein Class and Reactome Pathway analysis of 1315 hepatocyte-specific HSF1 target genes; (**F**) PANTHER GO-Slim Molecular Function and PANTHER GO-Slim Biological Process analysis of 3676 spermatocyte-specific HSF1 target genes. Filled circles depict statistically significant enrichment; X axis shows -LOG10 (*p*-value) *p* < 0.05; Fisher’s exact test with Benjamini–Hochberg false discovery rate correction (FDR).

To support the existence of tissue-specific functions for HSF1, we compared HSF1 targets from three different murine (mouse) cell types, spermatocytes, oocytes, and hepatocytes. Similar to human tissues, we observed both ‘shared’ and cell type-specific HSF1 target genes in these cell types (Figure 3A, Tables S4 and S9). We identified numerous cell type-specific potential HSF1 targets: 3676 genes in spermatocytes, 48 genes in oocytes, and 1315 genes in hepatocytes. Interestingly, only five HSF1 target genes were ‘shared’ by the three cell types (*Ubc*, *Tex11*, *Msh3*, *Ubb,* and *Cit*). The reason for this is probably the high number of oocyte-specific HSF1 target genes, implying an oocyte-specific function for HSF1. Only 24 HSF1 targets identified in oocytes are ‘shared’ by at least two different cell types, suggesting that HSF1 functions in oocytes significantly differ from the general function of HSF1. Similar to human cell types, we observed a great number (1318; 20.72% of all targets) of ‘shared’ HSF1 targets in at least two different murine cell types (Figure 3A, Tables S4 and S9 ).

PANTHER gene set enrichment analysis (GSEA) of ‘shared’ mouse HSF1 targets revealed that most of the significantly enriched GO terms are related to the classical function of HSF1 in heat shock response (including *heat shock protein binding* (e.g., *Hspa1a*, *Ptges3*, *Hspa5,* and *Stip1*) and *cellular response to unfolded protein* (e.g., *Hspa8*, *Hspa9,* and *Hspa5*)] (Figure 3C, Tables S6 and S9). ‘Shared’ target genes were also enriched for biological process terms associated with RNA metabolism such as *RNA binding* (e.g., *Rpl4*, *Zfand2a*, *Setx*, *Srek1*, *Rbm27*, *Polr2d*, *Nop9*, *Celf1,* and *Slbp*), *ribosome biogenesis* (e.g., *Rpl7l1*, *Rpl7a*, *Rrp1b,* and *Xpo1*) and *mRNA processing* (e.g., *Sltm*, *Srrm4*, *Dusp11*, *Cpsf4,* and *Pum1*) (Figure 3C, Table 3, Tables S6 and S9). Many of these HSF targets have not been identified in a single-gene-analysis experiment, but high throughput analysis has predicted them, except for *Zfand2a*, as genes repressed directly by HSF1 (Table S6). It is quite intriguing that ‘shared’ HSF1 targets are enriched in several cell cycle checkpoint-associated terms such as *mitotic DNA integrity checkpoint* (e.g., *Topbp1*, *Orc1*, *Rad17,* and *Cdc6*) and *G2/M checkpoints* (*Rad1*, *Cdc6,* and *Ccnb2*), pointing to a role of HSF1 in the regulation of cell cycle (Figure 3C, Table 3, Tables S6 and S9).

Oocyte-specific HSF1 target genes were enriched in biological processes including sister chromatid segregation (e.g., Stag2, Bub1b, Stag3, and Ddx11), negative regulation of protein serine/threonine kinase activity (e.g., Cdkn1b, Dusp1, and Cdkn1c) and several Reactome pathways were associated with mitotic cell cycle regulation (Figure 3D, Tables S6 and S9). These results highlight a role for HSF1 in mitotic cell division and oogenesis.

In the spermatocyte-specific HSF1 target gene set, the most enriched term is *cell adhesion via plasma-membrane adhesion molecules*. HSF1 target genes associated with this GO term include those coding for cadherins (*Cdh4*, *Cdh19*, *Cdh15*, *Cdh26*, *Cdh2,* and *Cdh10*), nectins (*Nectin1* and *Nectin3*), and teneurin (*Tenm4*). This unexpected finding can be explained by the fact that samples used for Chip-seq in the source study were contaminated by round spermatids [35]. Round spermatids in turn are associated with Sertoli cells, suggesting that HSF1 may play a role in controlling Sertoli-germ cell adhesion or in sperm-oocyte interaction during fertilization (Figure 3E, Table 3, Tables S6 and S9). In the hepatocyte-specific HSF1 target gene set, a significant enrichment was detected in terms associated with RNA metabolism and hepatic functions such as Reactome pathway terms *mRNA splicing* (e.g., *Polr2g*, *Clp1*, *Hnrnp,l* and *Ddx23*) and *metabolism of vitamins and cofactors* (e.g., *Ttpa*, *Nt5e*, *Nnmt,* and *Slc5a6*) (Figure 3F, Tables S6 and S9).

### 2.6. Identifying HSF1 Target Genes Related to Ageing in Mouse

To identify putative HSF1 target genes involved in the ageing process of mice, we overlapped the mouse HSF1 target list with murine ageing-related genes provided by the GenAge database. We observed that there are 44 HSF1 targets (0.7% of all genes) associated with ageing in mouse (Figure 3B and Table S10). These genes involve components of the insulin/IGF1 signalling pathway (e.g., *Igf1r* and *Insr*), mTORC1-mediated signalling (e.g., *Atm* and *Mtor*), cellular stress response pathways (e.g., *Atm*, *Mtor*, *Tp53*, *Prdx1,* and *Sirt1*), and DNA damage repair (*Msh2*, *Neil1*, *Atm*, *Tp53bp1*, *Brca1*, *Xpa* and *Ercc4)* (Figure 3B and Table S10). The eight ageing-related ‘shared’ HSF1 targets include *Igf1r*, *Brca1*, *Sirt1*, *Top3b*, *Arhgap1*, *Xpa*, *NUDT1,* and *Trp53bp1*. Altogether, these results further support the role of HSF1 in the ageing process of mice.

### 2.7. Novel Conserved HSF1 Target Genes

**Figure 4:**
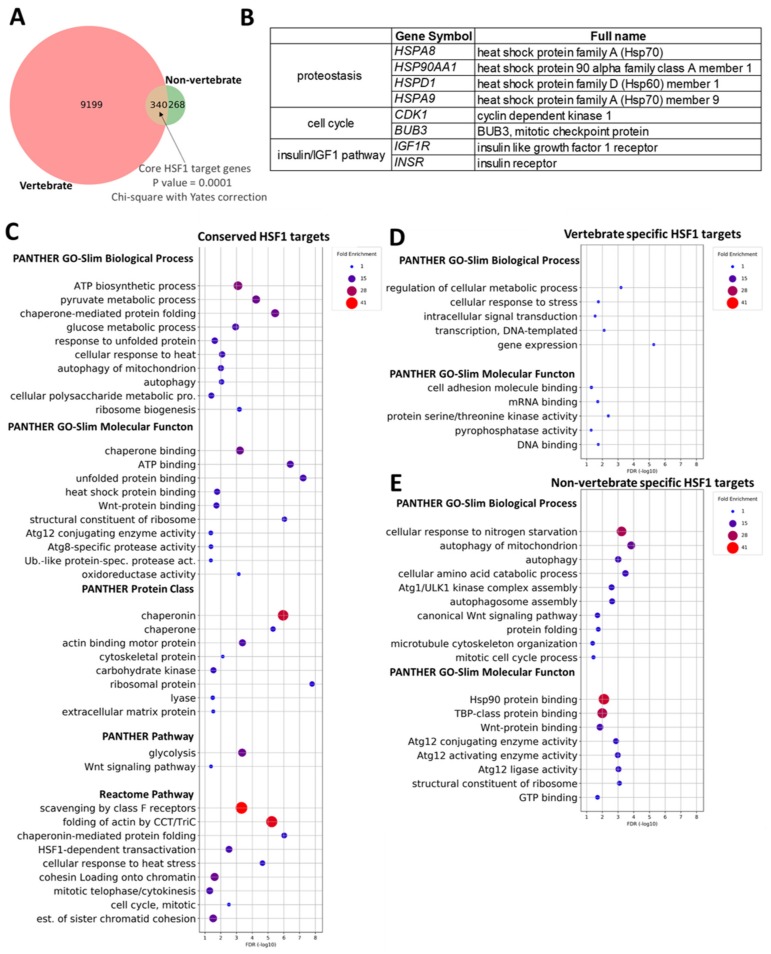
HSF1 targets are conserved between vertebrate and non-vertebrate species. (**A**) Venn-diagram depicting overlaps between HSF1 target genes in vertebrate (*H. sapiens, R. norvegicus,* and *M. musculus*) and non-vertebrate (*C. elegans, D. melanogaster,* and *S. cerevisiae*) species, (*p* = 0.0001, Chi-square with Yates correction; all unique vertebrate and non-vertebrate orthologues were used as background; 96,072 genes); (**B**) examples of HSF1 targets involved in ageing that are shared among groups vertebrate and non-vertebrate; (**C**) PANTHER GO-Slim Biological Process, PANTHER GO-Slim Molecular Function, PANTHER Protein Class, PANTHER Pathway, and Reactome Pathway analysis of 340 HSF1 target genes conserved between vertebrate and non-vertebrate species; (**D**) PANTHER GO-Slim Biological Process and PANTHER GO-Slim Molecular Function analysis of 9199 vertebrate-specific HSF1 target genes; (**E**) PANTHER GO-Slim Biological Process and PANTHER GO-Slim Molecular Function analysis of 268 non-vertebrate-specific HSF1 target genes. Filled circles depict statistically significant enrichment; X axis shows -LOG10 (*p*-value) *p* < 0.05; Fisher’s exact test with Benjamini–Hochberg False Discovery Rate correction (FDR).

To identify novel potential HSF1 target genes based on conservation throughout evolution, we first performed a cross-species analysis by determining the human orthologues of HSF1 targets in *S. cerevisiae*, *D. melanogaster*, *C. elegans*, *R. norvegicus,* and *M. musculus* (see Materials and Methods). In total, 9807 orthologous gene pairs were identified (Figure 4A and Table S5). Next, we divided these genes into two taxonomic subgroups: vertebrates and non-vertebrates. The vertebrate group contains human HSF1 targets and human orthologues of murine HSF1 target genes. The non-vertebrate group contains human orthologues of HSF1 target genes identified in worms, flies, and yeast. We found that some HSF1 target genes (340, 4% of vertebrate and 56% of non-vertebrate target genes) were ‘shared’ by the subgroups, while others were unique to a single subgroup; 9199 out of 9539 (96%) unique to vertebrate, and 268 out of 608 (44%) unique to non-vertebrate (Figure 4A and Table S5). 

The ‘shared’ HSF1 targets present in both subgroups were enriched for GO biological process terms associated with the classical role of HSF1, including proteostasis [like *chaperone-mediated protein folding* (e.g., *DNAJB5/dnj-13/SIS1*, *HSPA5/hsp-3/KAR2*, *HSPA8/hsp-1,* and *SGTB/SGT2*)], as well as terms such as *chaperone binding, chaperonin HSF1-dependent transactivation,* and *cellular response to heat stress* (Figure 4C, Tables S6 and S11). Interestingly, other significantly enriched HSF1 target genes are represented among biological process terms such as *mitophagy* (degradation of mitochondria by the lysosomal system) (*ATG2B/atg-2*, *FIS1/fis-1*, *ATG9A/atg-9*, *RB1CC1/epg-7,* and *ATG2A/atg-2*), *Atg8-specific protease activity* (*atg9a/atg-9*, *ATG101/epg-9*, *ATG2A/atg-2*, *atg2b/atg-2,* and *RB1CC1/epg-7*), *ATP biosynthetic process* (e.g., *PFKL/pkf-1.1*, *GAPDH/gpd-2*, *PFKM/pkf-1.1,* and *PFKP/pkf-1.1*), *ribosome biogenesis* (e.g., *RPL35A/rpl-33*, *RPS27/rps-27*, *SART1/F19F10.9*, *RPS7/rps-7*, *RPS21/rps-21*, *BTRC/lin-23,* and *RPS14/rps-14*), and molecular function terms such as *Wnt-protein binding* (e.g., *FZD1/mom-5*, *ROR2/cam-1* and *MIR4683/cfz-2*) (Figure 4C, Table 4, Tables S6 and S11). In many cases, the mouse orthologue of human-*C. elegans* orthologue pairs mentioned above was also found in the ‘shared’ target gene set (see for example, *Atg8-specific protease activity* (*ATG9A/Atg9a/atg-9*, *ATG101/Atg101/epg-9*, *ATG2A/Atg2a/atg-2,* and *ATG2B/Atg2b/atg-2*)) (Table S6). These results were consistent with the finding that ‘shared’ HSF1 target genes are enriched for PANTHER protein classes such as *ribosomal protein* and *extracellular matrix protein* (e.g., *LRIG2/sma-10*, *SART1/F19F10.9*, *SDC3/Y49E10.10,* and *ADAMTS1/T19D2.1*) and pathways such as *Wnt signalling* (e.g., *FZD1/mom-5*, *PRKCE/pkc-1*, *BTRC/lin-23,* and *PPP3CC/tax-6*) (Figure 4C, Table 4, Tables S6 and S11). Hence, HSF1 may play diverse conserved roles beyond its canonical function as a guardian of proteostasis.

Next, we analyzed HSF1 target genes being unique to each subgroup to find out whether HSF1 acquired or lost specific functions during evolution. PANTHER analysis of the Vertebrate HSF1 gene set showed only a moderate enrichment for specific terms (e.g., 1.89-fold enrichment in *cell adhesion molecule binding* consisting of 44 putative HSF1 target genes that encode proteins such as cadherins, nectins, teneurins, and plakophilins). We identified nearly half of these genes (26 out of 44) as HSF targets in both mice and human (Figure 4D, Tables S6 and S11). Nevertheless, the non-vertebrate gene set was significantly enriched in PANTHER terms such as *autophagy* (e.g., *lgg-1*, *lgg-2,* and *atg-18*) and *mitotic cell cycle process* (*mec-12*, *mec-7*, *T08D2.7,* and *R02F2.1*) in *C. elegans*. A closer look at the aforementioned autophagy-related genes revealed that they were directly inhibited by HSF-1 in *C. elegans* [9,12].

### 2.8. Ageing-Related HSF1 Target Genes Conserved Throughout Evolution

We further asked whether ‘shared’ orthologous targets in the groups vertebrate and non-vertebrate are associated with ageing. We found that out of the 340 ‘shared’ target genes, nine (2.6% of the ‘shared’ orthologue pairs) were related to ageing according to the GenAge database. These genes code for heat shock proteins (*HSPA8*, *HSP90AA1*, *HSPD1,* and *HSPA9*), regulators of cell cycle (*CDK1* and *BUB3*) components of insulin/IGF1 signalling (*INSR* and *IGF1R*) and the zinc metallopeptidase ZMPSTE24, highlighting the fact that HSF1 may play a role in ageing via maintaining cellular proteostasis and affecting the insulin/IGF1 pathway (Figure 4B and Table S12).

## 3. Discussion

In this work, we established a database called HSF1base that contains a comprehensive list of HSF1 target genes identified so far by single gene analyses and high throughput experiments. The PSI-MITAB 2.8 format makes the HSF1base to be an easy-to-use systems-level resource. The database provides 15,641 unique HSF1 gene interactions and contains 1321 HSF1-bound target genes, the expression of which is regulated in a HSF1-dependent manner (774 activated and 547 inhibited by the transcription factor). The target genes derive from several species such as *S. cerevisiae*, C. *elegans*, *D. melanogaster, M. musculus*, *R. norvegicus,* and *H. sapiens*. The HSF1base website (*hsf1base.org*) serves as a graphical interface and provides a user-friendly environment for the scientific community to interactively search, browse or download the database. The database was made in August 2019, and it will be updated regularly (in every half year).

Like most bioinformatics resources relying on high throughput data, the HSF1Base also has several limitations. Some of the HSF1 target gene interactions may not be functional or may function only under specific circumstances. This is caused by the fact that transcriptional activity of HSF1 highly depends on the nature and magnitude of cellular stress, as well as the type and actual state (cell cycle phase, metabolic, and differentiation status) of the affected cell [2,18,36]. It has been shown for example that HSF1 regulates different sets of target genes during cellular stress response, development, and tumorigenesis [12,15,16,37,38]. Thus, users should keep in mind that further experimental validation is required to confirm these interactions. Despite these limitations, useful information can be obtained for researchers working on the terrain of HSF1 biology, heat-shock proteins, and ageing.

According to the HSF1Base, a great number of HSF1 target genes (547 out of 1321) are down-regulated. This number is surprisingly high since a great majority of studies identified HSF1 as a transcriptional activator. In several publications, however, HSF1 has been described as a transcriptional repressor [39,40,41,42,43]. Moreover, in murine fibroblast cells HSF1 has been also shown to play a role in maintaining an open chromatin state in the proximity of *IL*-*6* gene, thereby endorsing the accessibility of other transcription factors to this regulatory region [44]. It is possible that HSF1 represses its target genes through modifying their chromatin structure. HSF1 was also reported to co-regulate a developmental program with E2F/DP transcription factors in *C. elegans* [12]. Based on this observation it is possible that HSF1 may repress genes in collaborating with other transcription factors.

To illustrate the applicability of the HSF1Base, we analyzed ‘shared’ and cell type-specific targets in human and murine cell types, using PANTHER. Our analysis showed that in both human and mouse cell types the main role of HSF1 is the regulation of proteostasis. This result is consistent with our general knowledge on HSF1 function, and supports relevant information on HSF1 functions that can be obtained using the HSF1Base. However, we also identified putative target genes shared by different cell types in human and mouse which play a role in diverse biological processes such as regulation of the circadian rhythm, chromatin modification, mitotic cell cycle, and RNA metabolism (Figure 1D and Figure 2C, Table 2 and Table S6).

HSF1 is induced upon cell stressors such as UV light, oxidative stress and heat stress, and triggers the synchronization of the circadian clock via directly regulating the core clock gene *Per2* [45,46,47]. According to the HSF1Base, some of the putative HSF1 targets associated with the circadian rhythm are upregulated (*RXRA*, *NOCT*, *BHLHE40*, *SERPINE1,* and *DBP*) while others are inhibited (*NCOA2*, *ARNTL,* and *TBL1X*) by the transcription factor (Figure 2D, Table 2 and Table S6). Among the eight targets listed above, only *SERPINE1* was previously described as a HSF1-regulated direct target gene [48].

It has been shown that after heat stress, HSF1 interacts with HDAC1 and HDCA2 histone deacetylases [49]. Thus, it may also function as a master regulator of stress-induced chromatin deacetylation. In good accordance with this assumption, here we identified several HSF1 targets which code for lysine (*KDM6A*, *KDM4B,* and *KDM1A*) or arginine (*JMJD6*) demethylases. HSF1 may thus directly activate *KDM4B* and *JMJD6* genes. Based on these data one may predict that HSF1 influences stress-induced chromatin reorganization via transcriptionally controlling the two genes. Indeed, HSF1 binds several (23) histone-related genes, two of which (*Hist2h2aa3* and *HIST2H2BE*) are directly upregulated by HSF1 (Figure 2D, Table 2 and Table S6).

Among the ‘shared’ human HSF1 targets, we identified several genes coding for extracellular and matrix structural proteins (*CRELD1*, *EFEMP1*, *LTBP4,* and *LTBP1*) (Figure 2D, Table 2 and Table S6). We suggest that HSF1 influences the process of extracellular matrix remodeling. HSF1 has been indeed identified as a key regulator of idiopathic pulmonary fibrosis [50]. Silencing of HSF1 impaired the expression of fibrillar collagen (*COL1A1* and *COL1A2*), and several genes required for the biogenesis of collagen fibers (*P4HA2*, *PLOD1,* and *FKBP4*) in pulmonary fibroblasts. HSF1 was also implicated in pulmonary fibroblasts to regulate extracellular matrix-specific gene expression [50].

We also identified cell type-specific roles for HSF1. In both human breast epithelium and murine spermatocyte (or round spermatids; see before), we found that a significant number of HSF1 target genes are related to cell adhesion (Figure 1G and Figure 2F, Table 3 and Table S6). Products of these target genes include cadherins, teneurins, and nectins, present in both breast epithelium and spermatocyte-specific HSF1 target gene sets. A role of HSF1 in cell adhesion and migration has been observed in several studies. In highly malignant human cancer cell lines, HSF1 activates genes involved in cell adhesion and migration [16]. Moreover, *FN1,* the gene coding for the extracellular matrix protein fibronectin, was recently identified as a stress-responsive gene regulated by HSF1 [51]. In hepatocellular carcinoma cells, HSF1 modulates E-Cadherin expression through directly regulating *Snail1* transcription [52]. Finally, in gastric cancer cells HSF1 overexpression stimulates vimentin, *N*-cadherin, and *Snail1* expression, and decreases the level of E-cadherin [53]. Taken together, it is plausible that HSF1 regulates *cadherin*, *teneurin,* and *nectin* genes directly in human breast epithelium.

In mammalian testis, cadherins, α-, β- or γ-catenin and nectins are important components in Sertoli–Sertoli and Sertoli-germ cell adhesion [54]. Teneurins playing a significant role in cell adhesion and migration are expressed predominantly in the central nervous system [55,56]. Nevertheless, a recent study showed that teneurins are also expressed in adult mouse testes and regulate testicular size and testosterone production [57]. Although the role of HSF1 in spermatogenesis has been studied extensively [17,37,58,59,60], involvement of HSF1-regulated genes encoding cell adhesion molecules in this process has not been described yet.

Comparison of HSF1 target genes in three murine cell types showed that most target genes were found exclusively in oocytes (Figure 3D, Table 3 and Table S6). This suggest a highly specific role for HSF1 during oogenesis. Indeed, in yeast HSF1 was identified as an essential checkpoint component required for mitotic progression [61]. Oocyte-specific functions of HSF1 were also described [38]. The result of gene ontology analysis performed in this study also showed that in oocytes HSF1 binds genes encoding key regulators of mitosis, cytokinesis, and core components of various signaling pathways. Taken together, our analysis supports that HSF1 functions can vary in the context of cell type.

We also analyzed the possible evolutionary conservation of putative HSF1 target genes. The assay revealed that beyond the HSR numerous HSF1 functions emerged across various eukaryotic taxa. According to our analysis, HSF1 targets involve for example several autophagy-related genes (Figure 4C, Table 4 and Table S6). Although regulatory interaction between HSF1 and autophagy has been well documented [62,63,64,65,66], *Atg* genes identified by our analysis as HSF1 targets (*ATG2B*, *FIS1*, *ATG9A*, *RB1CC1,* and *ATG2A*; see Figure 4C, Table S6) had not been examined for control by the transcription factor. Interestingly, our present study pointed to the fact that HSF1 indeed represses several *Atg* genes in *C. elegans*. These data were provided by two recent studies, in which the authors used high-throughput methods to uncover targets of CeHSF-1 (*C. elegans* HSF1) during development [12] and at adulthood followed by heat shock [9]. Both analyses explored the repression of several *Atg* genes (*lgg-1*, *lgg-2*, *atg-2*, *atg-9*, *atg-11,* and *atg-18*) by HSF-1.

In this study we showed that numerous putative HSF1 target genes associated with *ribosome biogenesis* term (e.g., *RPL35A/rpl-33*, *SART1/F19F10.9*, *RPS21/rps-21*, *RPL26/rpl-26,* and *HEATR3/ rpl-26*) are evolutionarily conserved between mice and nematodes (Figure 4C, Table 4 and Table S6). Moreover, we identified several ribosome biogenesis-associated genes as HSF1 targets in both mouse hepatocytes and spermatocytes (Figure 3E and Figure 2F, Table S7). According to the HSF1base, among these genes *RPL7L1* and *XPO1* are directly repressed by HSF1. Relationship between HSF1 activity and ribosome biogenesis has become clear in the last years only [67,68]. It is possible that in fast-growing cells HSF1 attenuates cellular stress triggered by orphan (not integrated into a ribosome) ribosomal proteins and rRNAs through inhibiting or activating ribosomal genes. 

The role of HSF1 in ageing has been well documented in *C. elegans* [69,70]. In other model organism, like *Drosophila*, overexpression of certain HSPs such as mitochondrial Hsp22 extends lifespan [71]. Furthermore, mutant mice defective for co-chaperone (CHIP) activity age faster than normal [72], and certain HSP-encoding genes become upregulated in long-lived mutant mouse strains [73]. Thus, it seems plausible that HSF1 influences ageing via controlling *HSP* gene activity. However, in *C. elegans* HSF1 affects also the activity of the longevity pathway TGFβ (transforming growth factor-beta) signaling [39], and autophagy [64]. Moreover, overexpressing a hypomorphic mutant allele of *hsf-1* that is not capable of inducing *hsp* genes also increases the survival of *C. elegans* [74]. In this study we identified several HSF1 targets that influence ageing independently of the HSR. Such genes encode for example a fibulin-like extracellular matrix protein (EFEMP1) [75,76] in different human tissues, a calcium voltage-gated channel subunit alpha1 A protein (CACNA1A) [77,78,79], and AP-1 transcription factor subunit JUN [80,81,82,83] (Figure 2B, Table S8). Among these factors, only *JUN* has been identified as a direct HSF1 target [29]. By comparing potential HSF1 targets in different mouse cell types we further identified eight genes that may modulate lifespan in a HSF1-dependent manner (Figure 3B, Table S10). The regulatory relationship between HSF1 and these potential targets has not been examined in single-gene-analysis studies. In addition, by monitoring the evolutionary conservation of potential HSF1 target genes we recognized five other genes that may modulate the rate at which cells age (Figure 4B, Table S12).

## 4. Materials and Methods

### 4.1. Origin of Datasets Used

Data for HSF1 target genes were obtained from 117 publications (see Table S1). PANTHER analysis of high throughput data was obtained from 14 publications (see Table S1).

### 4.2. Statistical Overrepresentation Tests 

Statistical overrepresentation tests (PANTHER GO-Slim Biological Process, PANTHER GO-Slim Molecular Function, PANTHER Protein Class, PANTHER Pathways and Reactome pathways) of gene sets were performed using the PANTHER database (http://PANTHERdb.org/, Accessed on 12 September 2019) [84]. Gene lists were uploaded in Ensemble Gene ID format, and default whole genome lists from the appropriate species were used as reference. To analyze statistical significance, Fisher’s exact test with Benjamini–Hochberg False Discovery Rate correction (FDR) was applied [85]. Over-represented terms were plotted by *Matplotlib* using *Python.*

### 4.3. Orthologue Analysis

*Mus musculus*, *Ratus norvegicus*, *Caenorhabditis elegans*, *Saccharomyces cerevisiae*, and *Drosophila melanogaster* orthologs of human genes were obtained from the *OMA orthology database* [86], using Python.

### 4.4. Venn Diagrams and Statistical Analysis of Overlaps

Venn diagrams were created in Python with *Matplotlib*. Statistical analysis for the significance of overlaps was performed using *super exact test* or *Chi*-square test with Yates correction in R [87]. For tests within a species, all unique genes within the given species were used as background. For the cross-species analysis, only the genes with orthologues (human–*C. elegans*, human–*D. melanogaster*, human–*R. norvegicus,* or human–*M. musculus* orthologues) were used as a background. 

## 5. Conclusions

In the last two decades a great number of novel functions were assigned to HSF1. The protein has been implicated in physiological and pathological processes such as cell cycle, apoptosis, circadian rhythm, immune response, as well as in ageing, and many age-related diseases, including cancer and neurodegenerative pathologies. The growing amount of high throughput data and the increasing interest in the scientific community on HSF1 justifies the development of a comprehensive, well-organized database of HSF1 targets. We believe that HSF1Base can be used as a resource to discover novel functions of HSF1.

## Figures and Tables

**Table 1:** HSF-1 targets found in the database HSF1base (*hsf1base.org*).

	Activated	Inhibited	N/D	All
	**ALL INTERACTIONS**			
All records	5258	1942	17,435	24,635
HSF1 targets with evidence for direct interaction	577	344	17,435	18,356
HSF1 targets with evidence for HSF1 dependent regulation	4681	1598	0	6279
HSF1 targets with evidence for direct interaction and HSF1 dependent regulation	1869	1023	0	2892
	**UNIQUE INTERACTIONS**			
All records	3869	1272	10,500	15,641
HSF1 targets with evidence for direct interaction	404	207	10,499	11,110
HSF1 targets with evidence for HSF1 dependent regulation	3877	1325	0	5202
HSF1 targets with evidence for direct interaction and HSF1 dependent regulation	774	547	0	1321
	**ALL INTERACTIONS**			
*Homo sapiens*	1685	958	7955	10,598
*Mus musculus*	135	279	9315	9729
*Rattus norvegicus*	465	166	0	631
*Caenorhabditis elegans*	2662	492	0	3154
*Drosophila melanogaster*	99	38	54	191
*Saccharomyces cerevisiae*	211	5	111	327
other	0	3	0	3
	**UNIQUE INTERACTIONS**			
*Homo sapiens*	876	562	4182	5620
*Mus musculus*	83	143	6153	6379
*Rattus norvegicus*	454	146	0	600
*Caenorhabditis elegans*	2218	375	0	2593
*Drosophila melanogaster*	98	38	54	190
*Saccharomyces cerevisiae*	140	5	111	256
other	0	3	0	3

A table summarizing the HSF-1 targets found in the database HSF1base (*hsf1base.org*). The ‘activated’ gene expression is upregulated by HSF-1. The ‘inhibited’ gene expression is downregulated by HSF-1. ‘N/D’ means there is no regulatory information available. ‘All interactions’ contains all entries in the database; duplicates are included. ‘Unique interactions’ does not contain duplicates; every gene is included only once. ‘HSF1 targets with evidence for direct interaction’ are considered as such based on evidence for physical interaction between HSF1 and the corresponding gene. In case of ‘HSF1 targets with evidence for HSF1 dependent regulation’, regulatory information is available, but there is no evidence for physical interaction between HSF1 and the corresponding gene. For ‘HSF1 targets with evidence for direct interaction and HSF1 dependent regulation’, both regulatory information and evidence for physical interaction are available.

**Table 2:** Selected list of HSF1 target genes identified in human cell types, using the HSF1Base.

Human ’Core’ HSF1 Target Genes		
**Extracellular matrix structural protein**		
**Official Gene Symbol**	**Novelty (target not characterized in detail)**	**Regulatory information**
*creld1*	Yes	activated
*EFEMP1*	Yes	activated
*LTBP4*	Yes	activated
**Signaling by NOTCH1**		
**Official Gene Symbol**	**Novelty (target not characterized in detail)**	**Regulatory information**
*UBB*	No	activated
*UBC*	Yes	activated
*notch1*	Yes	activated
*TBL1X*	Yes	inhibited
*DTX2*	Yes	activated
**Apoptotic pathway**		
**Official Gene Symbol**	**Novelty (target not characterized in detail)**	**Regulatory information**
*HSPA1L*	Yes	activated
*HSPA1A*	No	activated
*hspa1b*	No	activated
*JUN*	No	activated
*HSPA6*	Yes	activated
*BCL2L1*	Yes	activated
*FOS*	Yes	activated
*ATF3*	No	activated
*BAG3*	No	activated
*HSPA8*	Yes	activated
*MAP4K1*	Yes	activated
*NFKB2*	Yes	activated
*CFLAR*	Yes	activated
**Human ’shared’ HSF1 target genes**		
**Inflammasomes**		
**Official Gene Symbol**	**Novelty (target not characterized in detail)**	**Regulatory information**
*BCL2L1*	Yes	activated
*SUGT1*	Yes	activated
*APP*	Yes	activated
*MEFV*	Yes	activated
*HSP90AB1*	Yes	activated
*NFKB2*	Yes	activated
**BMAL1:CLOCK,NPAS2 activates circadian gene expression**		
**Official Gene Symbol**	**Novelty (target not characterized in detail)**	**Regulatory information**
*RXRA*	Yes	activated
*NOCT*	Yes	activated
*NCOA2*	Yes	inhibited
*BHLHE40*	Yes	activated
*ARNTL*	Yes	inhibited
*SERPINE1*	No	activated
*DBP*	Yes	activated
*TBL1X*	Yes	inhibited
**RAF-independent MAPK1/3 activation**		
**Official Gene Symbol**	**Novelty (target not characterized in detail)**	**Regulatory information**
*DUSP2*	Yes	activated
*DUSP10*	Yes	activated
*DUSP5*	Yes	activated
*MAP2K2*	Yes	activated
*DUSP1*	Yes	activated
**HDMs demethylate histones**		
**Official Gene Symbol**	**Novelty (target not characterized in detail)**	**Regulatory information**
*PHF8*	Yes	activated
*ARID5B*	Yes	activated
*KDM4B*	Yes	activated
*JMJD6*	Yes	activated

A table summarizing the HSF1 target genes identified using the database HSF1base. Selected HSF1 target genes are ordered by functional categories, based on PANTHER analyses. A target gene is considered novel if it had not previously been characterized as a HSF1 target in a single-gene-analysis study. Regulatory information is ‘activated’ or ‘inhibited’ if there is evidence for HSF1 dependent up- or down-regulation of the expression of the given gene. ‘N/D’ means there is no regulatory information available.

**Table 3:** Selected list of HSF1 target genes identified in murine cell types, using the HSF1Base.

Murine ’Shared’ HSF1 Target Genes			
**Ribosome biogenesis**			
**Official Gene Symbol**	**Novelty (target not characterized in detail)**	**Regulatory information**	
*RPL7L1*	Yes	inhibited	
*XPO1*	Yes	inhibited	
**G2/M Checkpoints**			
**Official Gene Symbol**	**Novelty (target not characterized in detail)**	**Regulatory information**	
*TOPBP1*	Yes	inhibited	
*RAD1*	Yes	inhibited	
*UBC*	No	activated	
**Oocyte**			
**Sister chromatid segregation**			
**Official Gene Symbol**	**Novelty (target not characterized in detail)**	**Regulatory information**	
*STAG3*	Yes	inhibited	
*STAG2*	Yes	activated	
**Spermatocyte**			
**Cell–cell adhesion via plasma-membrane adhesion molecules**			
**Official Gene Symbol**	**Novelty (target not characterized in detail)**	**Regulated directly by HSF1**	**Regulatory information**
*CRB1*	Yes	N/D	N/D
*TENM4*	Yes	N/D	N/D
*CDH19*	Yes	N/D	N/D
*CCDC141*	Yes	N/D	N/D
*CDH12*	Yes	N/D	N/D
*CDH18*	Yes	N/D	N/D
*SDK1*	Yes	N/D	N/D
*CNTN5*	Yes	N/D	N/D
*CDH6*	Yes	N/D	N/D
*CDH26*	Yes	N/D	N/D
*NECTIN1*	Yes	N/D	N/D
*CDH15*	Yes	N/D	N/D
*CDH13*	Yes	N/D	N/D
*CNTN6*	Yes	N/D	N/D
*CDH2*	Yes	N/D	N/D
*NECTIN3*	Yes	N/D	N/D
*CDH10*	Yes	N/D	N/D
*MYPN*	Yes	N/D	N/D
*CDH4*	Yes	N/D	N/D

A table summarizing the HSF1 target genes identified, using the database HSF1base. Selected HSF1 target genes are ordered by functional categories, based on PANTHER analyses. A target gene is considered novel if it had not previously been characterized as a HSF1 target in a single-gene-analysis study. Regulatory information is ‘activated’ or ‘inhibited’ if there is evidence for HSF1 dependent up- or down-regulation of the expression of the given gene. ‘N/D’ means there is no regulatory information available.

**Table 4:** Selected list of HSF1 target genes conserved between vertebrate and non-vertebrate species, identified by using HSF1Base.

HSF1 Target Genes Conserved between Vertebrate and Non-Vertebrate Species											
**Human**		**Mouse**		**Rat**		**Worm**		**Fly**		**Yeast**	
**autophagy**											
**Off. gene symb.**	**Reg. info.**	**Off. gene symb.**	**Reg. info.**	**Off. gene symb.**	**Reg. info.**	**Off. gene symb.**	**Reg. info.**	**Off. gene symb.**	**Reg. info.**	**Off. gene symb.**	**Reg. info.**
*FIS1*	N/D	N/D	N/D	N/D	N/D	*fis-1*	N/D	N/D	N/D	N/D	N/D
*atg9a*	N/D	*atg9a*	N/D	*atg9a*	N/D	*atg-9*	N/D	N/D	N/D	N/D	N/D
*ATG2A*	N/D	*ATG2A*	N/D	N/D	N/D	*atg-2*	N/D	N/D	N/D	N/D	N/D
*HSPA8*	act.	*HSPA8*	act.	N/D	N/D	*HSP-1*	act.	N/D	N/D	N/D	N/D
*atg2b*	N/D	*atg2b*	N/D	N/D	N/D	*atg-2*	N/D	N/D	N/D	N/D	N/D
*RB1CC1*	N/D	N/D	N/D	N/D	N/D	*epg-7*	N/D	N/D	N/D	N/D	N/D
**ribosome biogenesis**											
**Off. gene symb.**	**Reg. info.**	**Off. gene symb.**	**Reg. info.**	**Off. gene symb.**	**Reg. info.**	**Off. gene symb.**	**Reg. info.**	**Off. gene symb.**	**Reg. info.**	**Off. gene symb.**	**Reg. info.**
*RPL35A*	N/D	*RPL35A*	N/D	N/D	N/D	*rpl-33*	N/D	N/D	N/D	N/D	N/D
*RPS27*	N/D	N/D	N/D	N/D	N/D	*rps-27*	N/D	N/D	N/D	N/D	N/D
*SART1*	N/D	*SART1*	N/D	N/D	N/D	*F19F10.9*	N/D	N/D	N/D	N/D	N/D
*RPS7*	N/D	N/D	N/D	N/D	N/D	*rps-7*	N/D	N/D	N/D	N/D	N/D
*RPS21*	N/D	*RPS21*	N/D	N/D	N/D	*rps-21*	N/D	N/D	N/D	N/D	N/D
*BTRC*	N/D	N/D	N/D	N/D	N/D	*lin-23*	N/D	N/D	N/D	N/D	N/D
*RPS14*	act.	N/D	N/D	N/D	N/D	*rps-14*	N/D	N/D	N/D	N/D	N/D
*RPL26*	N/D	*RPL26*	N/D	N/D	N/D	*rpl-26*	N/D	N/D	N/D	N/D	N/D
*HEATR3*	N/D	*HEATR3*	N/D	N/D	N/D	N/D	N/D	*CG10286*	act.	N/D	N/D
*MRTO4*	N/D	*N/D*	N/D	*MRTO4*	N/D	N/D	N/D	N/D	N/D	*MRT4*	act.
*RPL26L1*	N/D	*RPL26*	N/D	N/D	N/D	*rpl-26*	N/D	N/D	N/D	N/D	N/D
**Glycolysis**											
**Off. gene symb.**	**Reg. info.**	**Off. gene symb.**	**Reg. info.**	**Off. gene symb.**	**Reg. info.**	**Off. gene symb.**	**Reg. info.**	**Off. gene symb.**	**Reg. info.**	**Off. gene symb.**	**Reg. info.**
*PGAM1*	N/D	*PGAM1*	N/D	N/D	N/D	N/D	N/D	N/D	N/D	*GPM1*	act.
*GPI*	N/D	*Gpi1*	N/D	N/D	N/D	N/D	N/D	*Pgi*	act.	N/D	N/D
*PGK1*	N/D	*PGK1*	N/D	N/D	N/D	N/D	N/D	N/D	N/D	*PGK1*	act.
**Wnt signalling pathway**											
**Off. gene symb.**	**Reg. info.**	**Off. gene symb.**	**Reg. info.**	**Off. gene symb.**	**Reg. info.**	**Off. gene symb.**	**Reg. info.**	**Off. gene symb.**	**Reg. info.**	**Off. gene symb.**	**Reg. info.**
*SMAD1*	N/D	*SMAD1*	inh.	N/D	N/D	*sma-2*	N/D	N/D	N/D	N/D	N/D
*PPP3CB*	N/D	*PPP3CB*	inh.	N/D	N/D	*tax-6*	N/D	N/D	N/D	N/D	N/D
*FBXW11*	inh.	*FBXW11*	N/D	N/D	N/D	*lin-23*	N/D	N/D	N/D	N/D	N/D
**Establishment of Sister Chromatid Cohesion**											
**Off. gene symb.**	**Reg. info.**	**Off. gene symb.**	**Reg. info.**	**Off. gene symb.**	**Reg. info.**	**Off. gene symb.**	**Reg. info.**	**Off. gene symb.**	**Reg. info.**	**Off. gene symb.**	**Reg. info.**
*SMC3*	N/D	*SMC3*	N/D	N/D	N/D	N/D	N/D	N/D	N/D	*SMC3*	act.

A table summarizing the HSF1 target genes identified by using the database HSF1base. Selected HSF1 target genes are ordered by functional categories based on PANTHER analyses. A target gene is considered novel if it had not previously been characterized as a HSF1 target in a single-gene-analysis study. ‘Off. gene symb.’ stands for official gene symbol. ‘Reg. info.’ stands for regulatory information. ‘act.’ stands for activated, ‘inh.’ stands for inhibited. Regulatory information is activated or inhibited if there is evidence for HSF1 dependent up- or down-regulation of the expression of the given gene. ‘N/D’ means there is no regulatory information available.

## Supplementary Materials

The following are available online at https://www.mdpi.com/1422-0067/20/22/5815/s1. Table S1: List of publications used to establish the database. ‘Relevant publications on individual HSF1 target genes’: List of HSF1-related publications on single gene analyses used. ‘Publications containing high throughput data’: List of publications HSF1-related publications containing high-throughput data used. Table S2: List of HSF1 target genes found in the database. ‘Statistics’: Table summarizing the HSF-1 targets found in Table S2. ‘Putative HSF1 targets’: List of all target genes included in ‘Unique interactions’ (see: Table 1). ‘Direct HSF1 targets’: List of ‘HSF1 targets with evidence for direct interaction’ included in ‘Unique interactions’ (see: Table 1). ‘Targets with HSF1 dep. expr’ (Targets with HSF1 dependent expression): List of ‘HSF1 targets with evidence for HSF1 dependent regulation’ included in ‘Unique interactions’ (see: Table 1). ‘Directly reg. HSF1 targets 1’ (directly regulated HSF1 targets 1): List of ‘HSF1 targets with evidence for direct interaction and HSF1 dependent regulation’ in a single publication (see: Table 1). ‘Directly reg. HSF1 targets 2’ (directly regulated HSF1 targets 2): List of ‘HSF1 targets with evidence for direct interaction and HSF1 dependent regulation’ in a single publication. The evidence for physical interaction between HSF1 and the gene and the regulatory information came from two separate publications (see: Table 1). ‘Directly regulated HSF1 targets’: The union of ‘Directly reg. HSF1 targets 1’ and ‘Directly reg. HSF1 targets 2’. The equivalent of the list of ‘HSF1 targets with evidence for direct interaction and HSF1 dependent regulation’ (see: Table 1). Table S3: HSF1 target genes in human by tissue/cell type. ‘Core targets’: List of HSF1 target genes shared between all three examined cell types in human. ‘Shared targets’: List of HSF1 target genes shared between at least two examined cell types in human. ‘Cervix adenocarcinoma’: List of cervix adenocarcinoma specific HSF1 targets in human. ‘Erythroleukemia cells’: List of erythroleukemia cells specific HSF1 targets in human. ‘Breast epithelium’: List of breast epithelium specific HSF1 targets in human. Table S4: HSF1 target genes in mouse by tissue/cell type. ‘Core targets’: List of HSF1 target genes shared between all three examined cell types in mouse. ‘Shared targets’: List of HSF1 target genes shared between at least two examined cell types in mouse. ‘Oocyte’: List of oocyte specific HSF1 targets in mouse. ‘Spermatocyte’: List of spermatocyte specific HSF1 targets in mouse. ‘Hepatocyte’: List of hepatocyte specific HSF1 targets in mouse. Table S5: Conserved and unique HSF1 target genes in groups vertebrate and non-vertebrate. ‘Vertebrate_Nonvertebrete_shared’: List of HSF1 target genes shared between the examined vertebrate and non-vertebrate species. ‘Vertebrate_unique’: List of HSF1 target genes only found in the examined vertebrate species. ‘Nonvertebrate_unique’: List of HSF1 target genes only found in the examined non-vertebrate species. Table S6: Selected HSF1 target genes over-represented in GO categories. ‘Selected HSF1 targets in human’: List of selected putative HSF1 target genes over-represented in GO categories in human. ‘Selected HSF1 targets in mouse’: List of selected putative HSF1 target genes over-represented in GO categories in mouse. ‘Selected conserved HSF1 targets’: List of selected conserved HSF1 target genes over-represented in GO categories shared between the examined vertebrate and non-vertebrate species. ‘Selected HSF1 targets in Verteb’ (Selected HSF1 targets in Vertebrate): List of selected conserved HSF1 target genes over-represented in GO categories in the examined vertebrate species. ‘HSF1 targets in Non-vertebrate’: List of selected conserved HSF1 target genes over-represented in GO categories in the examined non-vertebrate species. Table S7: PANTHER analyses of human HSF1 target genes by tissue/cell type. ‘Core targets’: PANTHER analyses of human HSF1 target genes shared between all three examined cell types. ‘Shared targets’: PANTHER analyses of human HSF1 target genes shared between at least two examined cell types. ‘Adenocarcinoma’: PANTHER analyses of cervix adenocarcinoma specific human HSF1 target genes. ‘Erythroleukemia’: PANTHER analyses of erythroleukemia specific human HSF1 target genes. ‘Breast epithelium’: PANTHER analyses of breast epithelium specific human HSF1 target genes. Table S8: HSF1 target genes related to ageing in human. ‘All human HSF1 targets’: List of all human HSF1 target genes related to ageing according to the GenAge database. ‘Directly regulated HSF1 targets’: List of human ‘HSF1 targets with evidence for direct interaction and HSF1 dependent regulation’ (see: Table 1) according to the GenAge database. Table S9: PANTHER analyses of mouse HSF1 target genes by tissue / cell type. ‘Core targets’: PANTHER analyses of mouse HSF1 target genes shared between all three examined cell types. ‘Shared targets’: PANTHER analyses of mouse HSF1 target genes shared between at least two examined cell types. ‘Oocyte’: PANTHER analyses of oocyte specific mouse HSF1 target genes. ‘Spermatocyte’: PANTHER analyses of spermatocyte specific mouse HSF1 target genes. ‘Hepatocyte’: PANTHER analyses of hepatocyte specific mouse HSF1 target genes. Table S10: HSF-1 target genes related to ageing in mice. ‘All mouse HSF1 targets’: List of all mouse HSF1 target genes related to ageing according to the GenAge database. ‘Directly regulated HSF1 targets’: List of mouse ‘HSF1 targets with evidence for direct interaction and HSF1 dependent regulation’ (see: Table 1) according to the GenAge database. Table S11: PANTHER analyses of conserved and unique HSF1 target genes in groups vertebrate and non-vertebrate. ‘Vertebrate’: PANTHER analyses of conserved and unique HSF1 target genes in the examined vertebrate species. ‘Non-vertebrate’: PANTHER analyses of conserved and unique HSF1 target genes in the examined non-vertebrate species. Table S12: HSF1 target genes related to ageing by organism and tissue/cell type. ‘Human’: List of HSF1 target genes related to ageing in human. ‘Mouse’: List of HSF1 target genes related to ageing in mouse. ‘Worm’: List of HSF1 target genes related to ageing in *C. elegans*. ‘Fly’: List of HSF1 target genes related to ageing in *D. melanogaster*. ‘Yeast’: List of HSF1 target genes related to ageing in *S. cerevisiae*.
