# Supplementary material for: HSF1Base: A Comprehensive Database of HSF1 (Heat Shock Factor 1) Target Genes
Source: Int J Mol Sci. 2019 Nov 19;20(22):5815. doi: 10.3390/ijms20225815 (PMC6888953; doi:10.3390/ijms20225815)
Supplement: Supplementary file 1 [file ijms-20-05815-s001.zip › Table_S1.pdf]

Relevant publications on individual HSF1 target genes			
**Publication ID**	**First author**	**Species**	**Reference**
pubmed:26759377	Joo et al.(2016)	*Caenorhabditis elegans*	[1]
pubmed:21408062	Iser et al.(2011)	*Caenorhabditis elegans*	[2]
pubmed:23116063	Barna et al.(2012)	*Caenorhabditis elegans*	[3]
pubmed:25324391	Baird et al.(2014)	*Caenorhabditis elegans*	[4]
pubmed:20351174	Ferguson et al.(2010)	*Caenorhabditis elegans*	[5]
pubmed:12468538	Xie et al.(2003)	*Cricetulus griseus*	[6]
pubmed:6472466	Wu et al.(1984)	*Drosophila melanogaster*	[7]
pubmed:6722872	Parker et al.(1984)	*Drosophila melanogaster*	[8]
pubmed:3685975	Wu et al.(1987)	*Drosophila melanogaster*	[9]
pubmed:4028160	Topol et al.(1985)	*Drosophila melanogaster*	[10]
pubmed:25280995	Das et al.(2014)	*Homo sapiens*	[11]
pubmed: 28955905	Das et al.(2016)	*Homo sapiens*	[12]
pubmed:23180277	Jung et al.(2013)	*Homo sapiens*	[13]
pubmed:18286539	Franceschelli et al.(2008)	*Homo sapiens*	[14]
pubmed:19006120	Du et al.(2009)	*Homo sapiens*	[15]
pubmed:20692357	Song et al.(2010)	*Homo sapiens*	[16]
pubmed:20396959	Shunmei et al.(2010)	*Homo sapiens*	[17]
pubmed:20185824	Rossi et al.(2010)	*Homo sapiens*	[18]
pubmed:23386620	Desai et al.(2013)	*Homo sapiens*	[19]
pubmed:25905985	Luo et al.(2016)	*Homo sapiens*	[20]
pubmed:17259369	Zhao et al.(2007)	*Homo sapiens*	[21]
pubmed:20176657	Butcher et al.(2010)	*Homo sapiens*	[22]
pubmed:26157141	Lee et al.(2015)	*Homo sapiens*	[23]
pubmed:23959823	Yang et al.(2014)	*Homo sapiens*	[24]
pubmed:25355627	Ishikawa et al.(2015)	*Homo sapiens*	[25]
pubmed:24127737	Sawai et al.(2013)	*Homo sapiens*	[26]
pubmed:24361604	Sakurai et al.(2014)	*Homo sapiens*	[27]
pubmed:24465598	Das et al.(2014)	*Homo sapiens*	[28]
pubmed:1628823	Abravaya et al.(1992)	*Homo sapiens*	[29]
pubmed:20692357	Song et al.(2010)	*Homo sapiens*	[16]
pubmed:22669480	Ruckova et al.(2012)	*Homo sapiens*	[30]
pubmed:25053922	Ferat-Osorio et al.(2014)	*Homo sapiens*	[31]
pubmed:21266308	Maitiy et al.(2011)	*Homo sapiens*	[32]
pubmed:10328874	Housby et al.(1999)	*Homo sapiens*	[33]
pubmed:21459854	Rawat et al.(2011)	*Homo sapiens*	[34]
pubmed:19139882	Zhang et al.(2009)	*Homo sapiens*	[35]
pubmed:22347460	Rossi et al.(2012)	*Homo sapiens*	[36]
pubmed:19668225	Zhao et al.(2009)	*Homo sapiens*	[37]
pubmed:10816597	Vilaboa et al.(2000)	*Homo sapiens*	[38]
pubmed:14766217	Kim et al.(2004)	*Homo sapiens*	[39]
pubmed:11005379	Ibrahim et al.(2000)	*Homo sapiens*	[40]
pubmed:19337383	Singh et al.(2009)	*Homo sapiens*	[41]
pubmed:30307772	Ali et al.(2019)	*Homo sapiens*	[42]

pubmed:30305397	Lellahi et al.(2018)	*Homo sapiens*	[43]
pubmed:29733381	Grossi et al.(2018)	*Homo sapiens*	[44]
pubmed:29730197	Li et al.(2018)	*Homo sapiens*	[45]
pubmed:29339121	Tong et al.(2018)	*Homo sapiens*	[46]
pubmed:29247221	Dhanani et al.(2017)	*Homo sapiens*	[47]
pubmed:25435429	Toma-Jonik et al.(2015)	*Homo sapiens*	[48]
pubmed:24291777	Samarasinghe et al.(2014)	*Homo sapiens*	[49]
pubmed:27846364	Watanabe et al.(2016)	*Homo sapiens*	[50]
pubmed:23587601	Park et al.(2013)	*Homo sapiens*	[51]
pubmed:16382149	Tchénio et al.(2006)	*Homo sapiens*	[52]
pubmed:23192351	Dai et al.(2013)	*Mus musculus*	[53]
pubmed:21915348	Tamaru et al.(2011)	*Mus musculus*	[54]
pubmed:21455770	Zhang et al.(2011)	*Mus musculus*	[55]
pubmed:17766920	Inouye et al.(2007)	*Mus musculus*	[56]
pubmed:20018623	Takii et al.(2010)	*Mus musculus*	[57]
pubmed:15226319	Inouye et al.(2004)	*Mus musculus*	[58]
pubmed:22105779	Ambade et al.(2012)	*Mus musculus*	[59]
pubmed:11734555	Singh et al.(2002)	*Mus musculus*	[60]
pubmed:16306048	Takaki et al.(2006)	*Mus musculus*	[61]
pubmed:15483628	Fujimoto et al.(2004)	*Mus musculus*	[62]
pubmed:22549481	Zhang et al.(2012)	*Mus musculus*	[63]
pubmed:22129966	Rozenfeld et al.(2012)	*Mus musculus*	[64]
pubmed:17024176	Hayashida et al.(2006)	*Mus musculus*	[65]
pubmed:26344102	Ma et al.(2015)	*Mus musculus*	[66]
pubmed:22615365	Krishnamurthy et al.(2012)	*Mus musculus*	[67]
pubmed:21205885	Uchida et al.(2011)	*Mus musculus*	[68]
pubmed:25430510	Tan et al.(2015)	*Mus musculus*	[69]
pubmed:20802198	Akerfelt et al.(2010)	*Mus musculus*	[70]
pubmed:24123326	Feng et al.(2014)	*Mus musculus*	[71]
pubmed:24849358	Wang et al.(2014)	*Mus musculus*	[72]
pubmed:24504023	Jego et al.(2014)	*Mus musculus*	[73]
pubmed:30480104	Kawamura et al.(2018)	*Mus musculus*	[74]
pubmed:16083881	Hahn et al.(2005)	*Mus musculus*	[75]
pubmed:23636453	Welc et al.(2013)	*Mus musculus*	[76]
pubmed:22215620	Gabai et al.(2012)	*Mus musculus*	[77]
pubmed:24165036	Vydra et al.(2013)	*Mus musculus*	[78]
pubmed:24523537	Chen et al.(2014)	*Mus musculus*	[79]
pubmed:24954509	Chou et al.(2015)	*Mus musculus*	[80]
pubmed:16211253	Seo et al.(2005)	*Rattus norvegicus*	[81]
pubmed:22427437	Fan-xin et al.(2012)	*Rattus norvegicus*	[82]
pubmed:27194715	Luo et al.(2016)	*Rattus norvegicus*	[83]
pubmed:21827755	Sakurai et al.(2011)	*Saccharomyces cerevisiae*	[84]
pubmed:17586717	Yamamoto et al.(2007)	*Saccharomyces cerevisiae*	[85]
pubmed:16292667	Takemori et al.(2006)	*Saccharomyces cerevisiae*	[86]
pubmed:12399584	Lee et al.(2002)	*Saccharomyces cerevisiae*	[87]

pubmed:15343339	Harbison et al.(2004)	*Saccharomyces cerevisiae*	[88]
pubmed:16709784	Workman et al.(2006)	*Saccharomyces cerevisiae*	[89]
pubmed:20385592	Reimand et al.(2010)	*Saccharomyces cerevisiae*	[90]
pubmed:16926161	Eastmond et al.(2006)	*Saccharomyces cerevisiae*	[91]
pubmed:15647283	Yamamoto et al.(2005)	*Saccharomyces cerevisiae*	[92]
pubmed:16880382	Chua et al.(2006)	*Saccharomyces cerevisiae*	[93]
pubmed:30332327	Pincus et al.(2018)	*Saccharomyces cerevisiae*	[94]
pubmed:10411744	Boy-Marcotte et al.(1999)	*Saccharomyces cerevisiae*	[95]
pubmed:18070923	Erkina et al.(2008)	*Saccharomyces cerevisiae*	[96]
pubmed:9296388	Zarzov et al.(1997)	*Saccharomyces cerevisiae*	[97]
pubmed:24237754	Hou et al.(2014)	*Saccharomyces cerevisiae*	[98]
pubmed:7739554	Giardina et al.(1995)	*Saccharomyces cerevisiae*	[99]
pubmed:8355706	Young et al.(1993)	*Saccharomyces cerevisiae*	[100]
pubmed:7651408	Halladay et al.(1995)	*Saccharomyces cerevisiae*	[101]
pubmed:17910533	Han et al.(2008)	*Saccharomyces cerevisiae*	[102]
pubmed:16556235	Hahn et al.(2006)	*Saccharomyces cerevisiae*	[103]

**Publications containing high througput data**			
**Publication ID**	**First author**	**Species**	**Reference**
pubmed:27688402	Li et al.(2016)	*Caenorhabditis elegans*	[104]
pubmed:27496166	Brunquell et al.(2016)	*Caenorhabditis elegans*	[105]
pubmed:30894454	Sural et al.(2019)	*Caenorhabditis elegans*	[106]
pubmed:15998452	Birch-Machin et al.(2005)	*Drosophila melanogaster*	[107]
pubmed:14668476	Trinklein et al.(2004)	*Homo sapiens*	[108]
pubmed:22863008	Mendillo et al.(2012)	*Homo sapiens*	[109]
pubmed:17216044	Page et al.(2006)	*Homo sapiens*	[110]
pubmed:23959860	Vihervaara et al.(2013)	*Homo sapiens*	[111]
pubmed:28369544	Vilaboa et al.(2017)	*Homo sapiens*	[112]
pubmed:21690297	Le Masson et al.(2011)	*Mus musculus*	[113]
pubmed:25450459	Korfanty et al.(2014)	*Mus musculus*	[114]
pubmed:23834426	Kus-Liśkiewicz et al.(2013)	*Mus musculus*	[115]
pubmed:30467350	Qu et al.(2018)	*Rattus norvegicus*	[116]
pubmed:15169889	Hahn et al.(2004)	*Saccharomyces cerevisiae*	[117]

Bibliography

1. Joo, H.-J.; Park, S.; Kim, K.-Y.; Kim, M.-Y.; Kim, H.; Park, D.; Paik, Y.-K. HSF-1 is involved in regulation of ascaroside pheromone biosynthesis by heat stress in Caenorhabditis elegans. *Biochem. J.* **2016**, *473*, 789–796.

2. Iser, W. B.; Wilson, M. A.; Wood, W. H.; Becker, K.; Wolkow, C. A. Co-regulation of the DAF-16 target gene, cyp-35B1/dod-13, by HSF-1 in C. elegans dauer larvae and daf-2 insulin pathway mutants. *PLoS One* **2011**, *6*, e17369.

3. Barna, J.; Princz, A.; Kosztelnik, M.; Hargitai, B.; Takács-Vellai, K.; Vellai, T. Heat shock factor-1 intertwines insulin/IGF-1, TGF-β and cGMP signaling to control development and aging. *BMC Dev. Biol.* **2012**, *12*, 32.

4. Baird, N. A.; Douglas, P. M.; Simic, M. S.; Grant, A. R.; Moresco, J. J.; Wolff, S. C.; Yates, J. R.; Manning, G.; Dillin, A. HSF-1-mediated cytoskeletal integrity determines thermotolerance and life span. *Science* **2014**, *346*, 360–363.

5. Ferguson, A. A.; Springer, M. G.; Fisher, A. L. skn-1-Dependent and -independent regulation of aip-1 expression following metabolic stress in Caenorhabditis elegans. *Mol. Cell. Biol.* **2010**, *30*, 2651–2667.

6. Xie, Y.; Zhong, R.; Chen, C.; Calderwood, S. K. Heat shock factor 1 contains two functional domains that mediate transcriptional repression of the c-fos and c-fms genes. *J. Biol. Chem.* **2003**, *278*, 4687–4698.

7. Wu, C. Activating protein factor binds in vitro to upstream control sequences in heat shock gene chromatin. *Nature* **1984**, *311*, 81–84.

8. Parker, C. S.; Topol, J. A Drosophila RNA polymerase II transcription factor binds to the regulatory site of an hsp 70 gene. *Cell* **1984**, *37*, 273–283.

9. Wu, C.; Wilson, S.; Walker, B.; Dawid, I.; Paisley, T.; Zimarino, V.; Ueda, H. Purification and properties of Drosophila heat shock activator protein. *Science* **1987**, *238*, 1247–1253.

10. Topol, J.; Ruden, D. M.; Parker, C. S. Sequences required for in vitro transcriptional activation of a Drosophila hsp 70 gene. *Cell* **1985**, *42*, 527–537.

11. Das, S.; Bhattacharyya, N. P. Heat shock factor 1 regulates hsa-miR-432 expression in human cervical cancer cell line. *Biochem. Biophys. Res. Commun.* **2014**, *453*, 461–466.

12. Das, S.; Bhattacharyya, N. P. Trans-activation of small EDRK-rich factor 2 (SERF2) promoter by Heat Shock Factor 1. *Biochemistry and Biophysics Reports* **2016**, *7*, 188–194.

13. Jung, M.-K.; Kim, K. Y.; Lee, N.-Y.; Kang, Y.-S.; Hwang, Y. J.; Kim, Y.; Sung, J.-J.; McKee, A.; Kowall, N.; Lee, J.; Ryu, H. Expression of taurine transporter (TauT) is modulated by heat shock factor 1 (HSF1) in motor neurons of ALS. *Mol. Neurobiol.* **2013**, *47*, 699–710.

14. Franceschelli, S.; Rosati, A.; Lerose, R.; De Nicola, S.; Turco, M. C.; Pascale, M. Bag3 gene expression is regulated by heat shock factor 1. *J. Cell Physiol.* **2008**, *215*, 575–577.

15. Du, Z.-X.; Zhang, H.-Y.; Meng, X.; Gao, Y.-Y.; Zou, R.-L.; Liu, B.-Q.; Guan, Y.; Wang, H.-Q. Proteasome inhibitor MG132 induces BAG3 expression through activation of heat shock factor 1. *J. Cell Physiol.* **2009**, *218*, 631–637.

16. Song, S.; Kole, S.; Precht, P.; Pazin, M. J.; Bernier, M. Activation of heat shock factor 1 plays a role in pyrrolidine dithiocarbamate-mediated expression of the co-chaperone BAG3. *Int. J. Biochem. Cell Biol.* **2010**, *42*, 1856–1863.

17. Shunmei, E.; Zhao, Y.; Huang, Y.; Lai, K.; Chen, C.; Zeng, J.; Zou, J. Heat shock factor 1 is a transcription factor of Fas gene. *Mol. Cells* **2010**, *29*, 527–531.

18. Rossi, A.; Trotta, E.; Brandi, R.; Arisi, I.; Coccia, M.; Santoro, M. G. AIRAP, a new human heat shock gene regulated by heat shock factor 1. *J. Biol. Chem.* **2010**, *285*, 13607–13615.

19. Desai, S.; Liu, Z.; Yao, J.; Patel, N.; Chen, J.; Wu, Y.; Ahn, E. E.-Y.; Fodstad, O.; Tan, M. Heat shock factor 1 (HSF1) controls chemoresistance and autophagy through transcriptional regulation of autophagy-related protein 7 (ATG7). *J. Biol. Chem.* **2013**, *288*, 9165–9176.

20. Luo, T.; Fu, J.; Xu, A.; Su, B.; Ren, Y.; Li, N.; Zhu, J.; Zhao, X.; Dai, R.; Cao, J.; Wang, B.; Qin, W.; Jiang, J.; Li, J.; Wu, M.; Feng, G.; Chen, Y.; Wang, H. PSMD10/gankyrin induces autophagy to promote tumor progression through cytoplasmic interaction with ATG7 and nuclear transactivation of ATG7 expression. *Autophagy* **2015**, *12*, 1–17.

21. Zhao, R.; Shen, G. X. Involvement of heat shock factor-1 in glycated LDL-induced upregulation of plasminogen activator inhibitor-1 in vascular endothelial cells. *Diabetes* **2007**, *56*, 1436–1444.

22. Butcher, N. J.; Minchin, R. F. Arylamine N-acetyltransferase 1 gene regulation by androgens requires a conserved heat shock element for heat shock factor-1. *Carcinogenesis* **2010**, *31*, 820–826.

23. Lee, J.-H.; Lee, Y.-K.; Lim, J. J.; Byun, H.-O.; Park, I.; Kim, G.-H.; Xu, W. G.; Wang, H.-J.; Yoon, G. Mitochondrial Respiratory Dysfunction Induces Claudin-1 Expression via Reactive Oxygen Species-mediated Heat Shock Factor 1 Activation, Leading to Hepatoma Cell Invasiveness. *J. Biol. Chem.* **2015**, *290*, 21421–21431.

24. Yang, X.; Wang, J.; Liu, S.; Yan, Q. HSF1 and Sp1 regulate FUT4 gene expression and cell proliferation in breast cancer cells. *J. Cell Biochem.* **2014**, *115*, 168–178.

25. Ishikawa, Y.; Sakurai, H. Heat-induced expression of the immediate-early gene IER5 and its involvement in the proliferation of heat-shocked cells. *FEBS J.* **2015**, *282*, 332–340.

26. Sawai, M.; Ishikawa, Y.; Ota, A.; Sakurai, H. The proto-oncogene JUN is a target of the heat shock transcription factor HSF1. *FEBS J.* **2013**, *280*, 6672–6680.

27. Sakurai, H.; Sawai, M.; Ishikawa, Y.; Ota, A.; Kawahara, E. Heat shock transcription factor HSF1 regulates the expression of the Huntingtin-interacting protein HYPK. *Biochim. Biophys. Acta* **2013**.

28. Das, S.; Bhattacharyya, N. P. Transcription regulation of HYPK by Heat Shock Factor 1. *PLoS One* **2014**, *9*, e85552.

29. Abravaya, K.; Myers, M. P.; Murphy, S. P.; Morimoto, R. I. The human heat shock protein hsp70 interacts with HSF, the transcription factor that regulates heat shock gene expression. *Genes Dev.* **1992**, *6*, 1153–1164.

30. Ruckova, E.; Muller, P.; Nenutil, R.; Vojtesek, B. Alterations of the Hsp70/Hsp90 chaperone and the HOP/CHIP co-chaperone system in cancer. *Cell Mol Biol Lett* **2012**, *17*, 446–458.

31. Ferat-Osorio, E.; Sánchez-Anaya, A.; Gutiérrez-Mendoza, M.; Boscó-Gárate, I.; Wong-Baeza, I.; Pastelin-Palacios, R.; Pedraza-Alva, G.; Bonifaz, L. C.; Cortés-Reynosa, P.; Pérez-Salazar, E.; Arriaga-Pizano, L.; López-Macías, C.; Rosenstein, Y.; Isibasi, A. Heat shock protein 70 down-regulates the

production of toll-like receptor-induced pro-inflammatory cytokines by a heat shock factor-1/constitutive heat shock element-binding factor-dependent mechanism. *J. Inflamm. (Lond.)* **2014**, *11*, 19.

32. Maity, T. K.; Henry, M. M.; Tulapurkar, M. E.; Shah, N. G.; Hasday, J. D.; Singh, I. S. Distinct, gene-specific effect of heat shock on heat shock factor-1 recruitment and gene expression of CXC chemokine genes. *Cytokine* **2011**, *54*, 61–67.

33. Housby, J. N.; Cahill, C. M.; Chu, B.; Prevelige, R.; Bickford, K.; Stevenson, M. A.; Calderwood, S. K. Non-steroidal anti-inflammatory drugs inhibit the expression of cytokines and induce HSP70 in human monocytes. *Cytokine* **1999**, *11*, 347–358.

34. Rawat, P.; Mitra, D. Cellular heat shock factor 1 positively regulates human immunodeficiency virus-1 gene expression and replication by two distinct pathways. *Nucleic Acids Res.* **2011**, *39*, 5879–5892.

35. Zhang, C.; Wang, Y.; Zhou, Z.; Zhang, J.; Tian, Z. Sodium butyrate upregulates expression of NKG2D ligand MICA/B in HeLa and HepG2 cell lines and increases their susceptibility to NK lysis. *Cancer Immunol. Immunother.* **2009**, *58*, 1275–1285.

36. Rossi, A.; Coccia, M.; Trotta, E.; Angelini, M.; Santoro, M. G. Regulation of cyclooxygenase-2 expression by heat: a novel aspect of heat shock factor 1 function in human cells. *PLoS One* **2012**, *7*, e31304.

37. Zhao, Y. H.; Zhou, M.; Liu, H.; Ding, Y.; Khong, H. T.; Yu, D.; Fodstad, O.; Tan, M. Upregulation of lactate dehydrogenase A by ErbB2 through heat shock factor 1 promotes breast cancer cell glycolysis and growth. *Oncogene* **2009**, *28*, 3689–3701.

38. Vilaboa, N. E.; Galán, A.; Troyano, A.; de Blas, E.; Aller, P. Regulation of multidrug resistance 1 (MDR1)/P-glycoprotein gene expression and activity by heat-shock transcription factor 1 (HSF1). *J. Biol. Chem.* **2000**, *275*, 24970–24976.

39. Kim, K.-Y.; Shin, S. M.; Kim, J. K.; Paik, S. G.; Yang, Y.; Choi, I. Heat shock factor regulates VDUP1 gene expression. *Biochem. Biophys. Res. Commun.* **2004**, *315*, 369–375.

40. Ibrahim, E. C.; Morange, M.; Dausset, J.; Carosella, E. D.; Paul, P. Heat shock and arsenite induce expression of the nonclassical class I histocompatibility HLA-G gene in tumor cell lines. *Cell Stress Chaperones* **2000**, *5*, 207–218.

41. Singh, U.; Bongcam-Rudloff, E.; Westermark, B. A DNA sequence directed mutual transcription regulation of HSF1 and NFIX involves novel heat sensitive protein interactions. *PLoS One* **2009**, *4*, e5050.

42. Ali, A.; Biswas, A.; Pal, M. HSF1 mediated TNF-α production during proteotoxic stress response pioneers proinflammatory signal in human cells. *FASEB J.* **2019**, *33*, 2621–2635.

43. Lellahi, S. M.; Rosenlund, I. A.; Hedberg, A.; Kiær, L. T.; Mikkola, I.; Knutsen, E.; Perander, M. The long noncoding RNA NEAT1 and nuclear paraspeckles are up-regulated by the transcription factor HSF1 in the heat shock response. *J. Biol. Chem.* **2018**, *293*, 18965–18976.

44. Grossi, V.; Forte, G.; Sanese, P.; Peserico, A.; Tezil, T.; Lepore Signorile, M.; Fasano, C.; Lovaglio, R.; Bagnulo, R.; Loconte, D. C.; Susca, F. C.; Resta, N.; Simone, C. The longevity SNP rs2802292 uncovered: HSF1 activates stress-dependent expression of FOXO3 through an intronic enhancer. *Nucleic Acids Res.* **2018**, *46*, 5587–5600.

45. Li, J.; Song, P.; Jiang, T.; Dai, D.; Wang, H.; Sun, J.; Zhu, L.; Xu, W.; Feng, L.; Shin, V. Y.; Morrison, H.; Wang, X.; Jin, H. Heat Shock Factor 1 Epigenetically Stimulates Glutaminase-1-Dependent mTOR Activation to Promote Colorectal Carcinogenesis. *Mol. Ther.* **2018**, *26*, 1828–1839.

46. Tong, Y.; Li, Y.; Gu, H.; Wang, C.; Liu, F.; Shao, Y.; Li, F. HSF1, in association with MORC2, downregulates ArgBP2 via the PRC2 family in gastric cancer cells. *Biochim. Biophys. Acta* **2018**.

47. Dhanani, K. C. H.; Samson, W. J.; Edkins, A. L. Fibronectin is a stress responsive gene regulated by HSF1 in response to geldanamycin. *Sci. Rep.* **2017**, *7*, 17617.

48. Toma-Jonik, A.; Widlak, W.; Korfanty, J.; Cichon, T.; Smolarczyk, R.; Gogler-Piglowska, A.; Widlak, P.; Vydra, N. Active heat shock transcription factor 1 supports migration of the melanoma cells via vinculin down-regulation. *Cell Signal.* **2015**, *27*, 394–401.

49. Samarasinghe, B.; Wales, C. T. K.; Taylor, F. R.; Jacobs, A. T. Heat shock factor 1 confers resistance to Hsp90 inhibitors through p62/SQSTM1 expression and promotion of autophagic flux. *Biochem. Pharmacol.* **2014**, *87*, 445–455.

50. Watanabe, Y.; Tsujimura, A.; Taguchi, K.; Tanaka, M. HSF1 stress response pathway regulates autophagy receptor SQSTM1/p62-associated proteostasis. *Autophagy* **2017**, *13*, 133–148.

51. Park, S.-J.; Sohn, H.-Y.; Park, S. I. TRAIL regulates collagen production through HSF1-dependent Hsp47 expression in activated hepatic stellate cells. *Cell Signal.* **2013**, *25*, 1635–1643.

52. Tchénio, T.; Havard, M.; Martinez, L. A.; Dautry, F. Heat shock-independent induction of multidrug resistance by heat shock factor 1. *Mol. Cell. Biol.* **2006**, *26*, 580–591.

53. Dai, B.; Gong, A.; Jing, Z.; Aldape, K. D.; Kang, S.-H.; Sawaya, R.; Huang, S. Forkhead box M1 is regulated by heat shock factor 1 and promotes glioma cells survival under heat shock stress. *J. Biol. Chem.* **2013**, *288*, 1634–1642.

54. Tamaru, T.; Hattori, M.; Honda, K.; Benjamin, I.; Ozawa, T.; Takamatsu, K. Synchronization of circadian Per2 rhythms and HSF1-BMAL1:CLOCK interaction in mouse fibroblasts after short-term heat shock pulse. *PLoS One* **2011**, *6*, e24521.

55. Zhang, L.; Yang, M.; Wang, Q.; Liu, M.; Liang, Q.; Zhang, H.; Xiao, X. HSF1 regulates expression of G-CSF through the binding element for NF-IL6/CCAAT enhancer binding protein beta. *Mol. Cell. Biochem.* **2011**, *352*, 11–17.

56. Inouye, S.; Fujimoto, M.; Nakamura, T.; Takaki, E.; Hayashida, N.; Hai, T.; Nakai, A. Heat shock transcription factor 1 opens chromatin structure of interleukin-6 promoter to facilitate binding of an activator or a repressor. *J. Biol. Chem.* **2007**, *282*, 33210–33217.

57. Takii, R.; Inouye, S.; Fujimoto, M.; Nakamura, T.; Shinkawa, T.; Prakasam, R.; Tan, K.; Hayashida, N.; Ichikawa, H.; Hai, T.; Nakai, A. Heat shock transcription factor 1 inhibits expression of IL-6 through activating transcription factor 3. *J. Immunol.* **2010**, *184*, 1041–1048.

58. Inouye, S.; Izu, H.; Takaki, E.; Suzuki, H.; Shirai, M.; Yokota, Y.; Ichikawa, H.; Fujimoto, M.; Nakai, A. Impaired IgG production in mice deficient for heat shock transcription factor 1. *J. Biol. Chem.* **2004**, *279*, 38701–38709.

59. Ambade, A.; Catalano, D.; Lim, A.; Mandrekar, P. Inhibition of heat shock protein (molecular weight 90 kDa) attenuates proinflammatory cytokines and prevents lipopolysaccharide-induced liver injury in mice. *Hepatology* **2012**, *55*, 1585–1595.

60. Singh, I. S.; He, J.-R.; Calderwood, S.; Hasday, J. D. A high affinity HSF-1 binding site in the 5'-untranslated region of the murine tumor necrosis factor-alpha gene is a transcriptional repressor. *J. Biol. Chem.* **2002**, *277*, 4981–4988.

61. Takaki, E.; Fujimoto, M.; Sugahara, K.; Nakahari, T.; Yonemura, S.; Tanaka, Y.; Hayashida, N.; Inouye, S.; Takemoto, T.; Yamashita, H.; Nakai, A. Maintenance of olfactory neurogenesis requires HSF1, a major heat shock transcription factor in mice. *J. Biol. Chem.* **2006**, *281*, 4931–4937.

62. Fujimoto, M.; Izu, H.; Seki, K.; Fukuda, K.; Nishida, T.; Yamada, S.-I.; Kato, K.; Yonemura, S.; Inouye, S.; Nakai, A. HSF4 is required for normal cell growth and differentiation during mouse lens development. *EMBO J.* **2004**, *23*, 4297–4306.

63. Zhang, H.; Zhang, L.; Yu, F.; Liu, Y.; Liang, Q.; Deng, G.; Chen, G.; Liu, M.; Xiao, X. HSF1 is a transcriptional activator of IL-10 gene expression in RAW264.7 macrophages. *Inflammation* **2012**, *35*, 1558–1566.

64. Rozenfeld, J.; Tal, O.; Kladnitsky, O.; Adler, L.; Efrati, E.; Carrithers, S. L.; Alper, S. L.; Zelikovic, I. The pendrin anion exchanger gene is transcriptionally regulated by uroguanylin: a novel enterorenal link. *Am. J. Physiol. Renal Physiol.* **2012**, *302*, F614–24.

65. Hayashida, N.; Inouye, S.; Fujimoto, M.; Tanaka, Y.; Izu, H.; Takaki, E.; Ichikawa, H.; Rho, J.; Nakai, A. A novel HSF1-mediated death pathway that is suppressed by heat shock proteins. *EMBO J.* **2006**, *25*, 4773–4783.

66. Ma, X.; Xu, L.; Alberobello, A. T.; Gavrilova, O.; Bagattin, A.; Skarulis, M.; Liu, J.; Finkel, T.; Mueller, E. Celastrol Protects against Obesity and Metabolic Dysfunction through Activation of a HSF1-PGC1α Transcriptional Axis. *Cell Metab.* **2015**, *22*, 695–708.

67. Krishnamurthy, K.; Vedam, K.; Kanagasabai, R.; Druhan, L. J.; Ilangovan, G. Heat shock factor-1 knockout induces multidrug resistance gene, MDR1b, and enhances P-glycoprotein (ABCB1)-based drug extrusion in the heart. *Proc. Natl. Acad. Sci. USA* **2012**, *109*, 9023–9028.

68. Uchida, S.; Hara, K.; Kobayashi, A.; Fujimoto, M.; Otsuki, K.; Yamagata, H.; Hobara, T.; Abe, N.; Higuchi, F.; Shibata, T.; Hasegawa, S.; Kida, S.; Nakai, A.; Watanabe, Y. Impaired hippocampal spinogenesis and neurogenesis and altered affective behavior in mice lacking heat shock factor 1. *Proc. Natl. Acad. Sci. USA* **2011**, *108*, 1681–1686.

69. Tan, J.; Tan, S.; Zheng, H.; Liu, M.; Chen, G.; Zhang, H.; Wang, K.; Tan, S.; Zhou, J.; Xiao, X. HSF1 functions as a transcription regulator for Dp71 expression. *Cell Stress Chaperones* **2015**, *20*, 371–379.

70. Akerfelt, M.; Vihervaara, A.; Laiho, A.; Conter, A.; Christians, E. S.; Sistonen, L.; Henriksson, E. Heat shock transcription factor 1 localizes to sex chromatin during meiotic repression. *J. Biol. Chem.* **2010**, *285*, 34469–34476.

71. Feng, Y.; Huang, W.; Meng, W.; Jegga, A. G.; Wang, Y.; Cai, W.; Kim, H. W.; Pasha, Z.; Wen, Z.; Rao, F.; Modi, R. M.; Yu, X.; Ashraf, M. Heat shock improves Sca-1+ stem cell survival and directs ischemic cardiomyocytes toward a prosurvival phenotype via exosomal transfer: a critical role for HSF1/miR-34a/HSP70 pathway. *Stem Cells* **2014**, *32*, 462–472.

72. Wang, X.; Cattaneo, F.; Ryno, L.; Hulleman, J.; Reixach, N.; Buxbaum, J. N. The Systemic Amyloid Precursor Transthyretin (TTR) Behaves as a Neuronal Stress Protein Regulated by HSF1 in SH-SY5Y Human Neuroblastoma Cells and APP23 Alzheimer's Disease Model Mice. *J. Neurosci.* **2014**, *34*, 7253–7265.

73. Jego, G.; Lanneau, D.; De Thonel, A.; Berthenet, K.; Hazoumé, A.; Droin, N.; Hamman, A.; Girodon, F.; Bellaye, P. S.; Wettstein, G.; Jacquel, A.; Duplomb, L.; Le Mouël, A.; Papanayotou, C.; Christians, E.; Bonniaud, P.; Lallemand-Mezger, V.; Solary, E.; Garrido, C. Dual regulation of SPI1/PU.1 transcription factor by heat shock factor 1 (HSF1) during macrophage differentiation of monocytes. *Leukemia* **2014**, *28*, 1676–1686.

74. Kawamura, G.; Hattori, M.; Takamatsu, K.; Tsukada, T.; Ninomiya, Y.; Benjamin, I.; Sassone-Corsi, P.; Ozawa, T.; Tamaru, T. Cooperative interaction among BMAL1, HSF1, and p53 protects mammalian cells from UV stress. *Commun. Biol.* **2018**, *1*, 204.

75. Hahn, J.-S. Regulation of Nod1 by Hsp90 chaperone complex. *FEBS Lett.* **2005**, *579*, 4513–4519.

76. Welc, S. S.; Judge, A. R.; Clanton, T. L. Skeletal muscle interleukin-6 regulation in hyperthermia. *Am. J. Physiol. Cell Physiol.* **2013**, *305*, C406–13.

77. Gabai, V. L.; Meng, L.; Kim, G.; Mills, T. A.; Benjamin, I. J.; Sherman, M. Y. Heat shock transcription factor Hsf1 is involved in tumor progression via regulation of hypoxia-inducible factor 1 and RNA-binding protein HuR. *Mol. Cell. Biol.* **2012**, *32*, 929–940.

78. Vydra, N.; Toma, A.; Glowala-Kosinska, M.; Gogler-Piglowska, A.; Widlak, W. Overexpression of Heat Shock Transcription Factor 1 enhances the resistance of melanoma cells to doxorubicin and paclitaxel. *BMC Cancer* **2013**, *13*, 504.

79. Chen, Y.; Wang, B.; Liu, D.; Li, J. J.; Xue, Y.; Sakata, K.; Zhu, L.; Heldt, S. A.; Xu, H.; Liao, F.-F. Hsp90 chaperone inhibitor 17-AAG attenuates Aβ-induced synaptic toxicity and memory impairment. *J. Neurosci.* **2014**, *34*, 2464–2470.

80. Chou, S.-D.; Murshid, A.; Eguchi, T.; Gong, J.; Calderwood, S. K. HSF1 regulation of β-catenin in mammary cancer cells through control of HuR/elavL1 expression. *Oncogene* **2015**, *34*, 2178–2188.

81. Seo, M. S.; Oh, S. Y.; Park, M. J.; Kim, S. M.; Kim, M. Y.; Han, S. I.; Park, H. G.; Kang, H. S. Implication of reactive oxygen species, ERK1/2, and p38MAPK in sodium salicylate-induced heat shock protein 72 expression in C6 glioma cells. *Int. J. Mol. Med.* **2005**, *16*, 841–849.

82. Fan-xin, M.; Li-mei, S.; Bei, S.; Xin, Q.; Yu, Y.; Yu, C. Heat shock factor 1 regulates the expression of the TRPV1 gene in the rat preoptic-anterior hypothalamus area during lipopolysaccharide-induced fever. *Exp. Physiol.* **2012**, *97*, 730–740.

83. Lou, Q.; Hu, Y.; Ma, Y.; Dong, Z. Heat shock factor 1 induces Crystallin-αB to protect against cisplatin nephrotoxicityα. *Am. J. Physiol. Renal Physiol.* **2016**, *311*, ajprenal.00201.2016.

84. Sakurai, H.; Ota, A. Regulation of chaperone gene expression by heat shock transcription factor in Saccharomyces cerevisiae: importance in normal cell growth, stress resistance, and longevity. *FEBS Lett.* **2011**, *585*, 2744–2748.

85. Yamamoto, A.; Ueda, J.; Yamamoto, N.; Hashikawa, N.; Sakurai, H. Role of heat shock transcription factor in Saccharomyces cerevisiae oxidative stress response. *Eukaryotic Cell* **2007**, *6*, 1373–1379.

86. Takemori, Y.; Sakaguchi, A.; Matsuda, S.; Mizukami, Y.; Sakurai, H. Stress-induced transcription of the endoplasmic reticulum oxidoreductin gene ERO1 in the yeast Saccharomyces cerevisiae. *Mol. Genet. Genomics* **2006**, *275*, 89–96.

87. Lee, T. I.; Rinaldi, N. J.; Robert, F.; Odom, D. T.; Bar-Joseph, Z.; Gerber, G. K.; Hannett, N. M.; Harbison, C. T.; Thompson, C. M.; Simon, I.; Zeitlinger, J.; Jennings, E. G.; Murray, H. L.; Gordon, D. B.; Ren, B.; Wyrick, J. J.; Tagne, J.-B.; Volkert, T. L.; Fraenkel, E.; Gifford, D. K.; Young, R. A. Transcriptional regulatory networks in Saccharomyces cerevisiae. *Science* **2002**, *298*, 799–804.

88. Harbison, C. T.; Gordon, D. B.; Lee, T. I.; Rinaldi, N. J.; Macisaac, K. D.; Danford, T. W.; Hannett, N. M.; Tagne, J.-B.; Reynolds, D. B.; Yoo, J.; Jennings, E. G.; Zeitlinger, J.; Pokholok, D. K.; Kellis, M.; Rolfe, P. A.; Takusagawa, K. T.; Lander, E. S.; Gifford, D. K.; Fraenkel, E.; Young, R. A. Transcriptional regulatory code of a eukaryotic genome. *Nature* **2004**, *431*, 99–104.

89. Workman, C. T.; Mak, H. C.; McCuine, S.; Tagne, J.-B.; Agarwal, M.; Ozier, O.; Begley, T. J.; Samson, L. D.; Ideker, T. A systems approach to mapping DNA damage response pathways. *Science* **2006**, *312*, 1054–1059.

90. Reimand, J.; Vaquerizas, J. M.; Todd, A. E.; Vilo, J.; Luscombe, N. M. Comprehensive reanalysis of transcription factor knockout expression data in Saccharomyces cerevisiae reveals many new targets. *Nucleic Acids Res.* **2010**, *38*, 4768–4777.

91. Eastmond, D. L.; Nelson, H. C. M. Genome-wide analysis reveals new roles for the activation domains of the Saccharomyces cerevisiae heat shock transcription factor (Hsf1) during the transient heat shock response. *J. Biol. Chem.* **2006**, *281*, 32909–32921.

92. Yamamoto, A.; Mizukami, Y.; Sakurai, H. Identification of a novel class of target genes and a novel type of binding sequence of heat shock transcription factor in Saccharomyces cerevisiae. *J. Biol. Chem.* **2005**, *280*, 11911–11919.

93. Chua, G.; Morris, Q. D.; Sopko, R.; Robinson, M. D.; Ryan, O.; Chan, E. T.; Frey, B. J.; Andrews, B. J.; Boone, C.; Hughes, T. R. Identifying transcription factor functions and targets by phenotypic activation. *Proc. Natl. Acad. Sci. USA* **2006**, *103*, 12045–12050.

94. Pincus, D.; Anandhakumar, J.; Thiru, P.; Guertin, M. J.; Erkine, A. M.; Gross, D. S. Genetic and epigenetic determinants establish a continuum of Hsf1 occupancy and activity across the yeast genome. *Mol. Biol. Cell* **2018**, *29*, 3168–3182.

95. Boy-Marcotte, E.; Lagniel, G.; Perrot, M.; Bussereau, F.; Boudsocq, A.; Jacquet, M.; Labarre, J. The heat shock response in yeast: differential regulations and contributions of the Msn2p/Msn4p and Hsf1p regulons. *Mol. Microbiol.* **1999**, *33*, 274–283.

96. Erkina, T. Y.; Tschetter, P. A.; Erkine, A. M. Different requirements of the SWI/SNF complex for robust nucleosome displacement at promoters of heat shock factor and Msn2- and Msn4-regulated heat shock genes. *Mol. Cell. Biol.* **2008**, *28*, 1207–1217.

97. Zarzov, P.; Boucherie, H.; Mann, C. A yeast heat shock transcription factor (Hsf1) mutant is defective in both Hsc82/Hsp82 synthesis and spindle pole body duplication. *J. Cell Sci.* **1997**, *110 (Pt 16)*, 1879–1891.

98. Hou, J.; Tang, H.; Liu, Z.; Österlund, T.; Nielsen, J.; Petranovic, D. Management of the endoplasmic reticulum stress by activation of the heat shock response in yeast. *FEMS Yeast Res* **2014**, *14*, 481–494.

99. Giardina, C.; Lis, J. T. Dynamic protein-DNA architecture of a yeast heat shock promoter. *Mol. Cell. Biol.* **1995**, *15*, 2737–2744.

100. Young, M. R.; Craig, E. A. *Saccharomyces cerevisiae* HSP70 heat shock elements are functionally distinct. *Mol. Cell. Biol.* **1993**, *13*, 5637–5646.

101. Halladay, J. T.; Craig, E. A. A heat shock transcription factor with reduced activity suppresses a yeast HSP70 mutant. *Mol. Cell. Biol.* **1995**, *15*, 4890–4897.

102. Han, Q.; Lu, J.; Duan, J.; Su, D.; Hou, X.; Li, F.; Wang, X.; Huang, B. Gcn5- and Elp3-induced histone H3 acetylation regulates hsp70 gene transcription in yeast. *Biochem. J.* **2008**, *409*, 779–788.

103. Hahn, J.-S.; Neef, D. W.; Thiele, D. J. A stress regulatory network for co-ordinated activation of proteasome expression mediated by yeast heat shock transcription factor. *Mol. Microbiol.* **2006**, *60*, 240–251.

104. Li, J.; Chauve, L.; Phelps, G.; Brielmann, R. M.; Morimoto, R. I. E2F coregulates an essential HSF developmental program that is distinct from the heat-shock response. *Genes Dev.* **2016**, *30*, 2062–2075.

105. Brunquell, J.; Morris, S.; Lu, Y.; Cheng, F.; Westerheide, S. D. The genome-wide role of HSF-1 in the regulation of gene expression in Caenorhabditis elegans. *BMC Genomics* **2016**, *17*, 559.

106. Sural, S.; Lu, T.-C.; Jung, S. A.; Hsu, A.-L. HSB-1 Inhibition and HSF-1 Overexpression Trigger Overlapping Transcriptional Changes to Promote Longevity in Caenorhabditis elegans. *G3 (Bethesda)* **2019**, *9*, 1679–1692.

107. Birch-Machin, I.; Gao, S.; Huen, D.; McGirr, R.; White, R. A. H.; Russell, S. Genomic analysis of heat-shock factor targets in Drosophila. *Genome Biol.* **2005**, *6*, R63.

108. Trinklein, N. D.; Murray, J. I.; Hartman, S. J.; Botstein, D.; Myers, R. M. The role of heat shock transcription factor 1 in the genome-wide regulation of the mammalian heat shock response. *Mol. Biol. Cell* **2004**, *15*, 1254–1261.

109. Mendillo, M. L.; Santagata, S.; Koeva, M.; Bell, G. W.; Hu, R.; Tamimi, R. M.; Fraenkel, E.; Ince, T. A.; Whitesell, L.; Lindquist, S. HSF1 drives a transcriptional program distinct from heat shock to support highly malignant human cancers. *Cell* **2012**, *150*, 549–562.

110. Page, T. J.; Sikder, D.; Yang, L.; Pluta, L.; Wolfinger, R. D.; Kodadek, T.; Thomas, R. S. Genome-wide analysis of human HSF1 signaling reveals a transcriptional program linked to cellular adaptation and survival. *Mol. Biosyst.* **2006**, *2*, 627–639.

111. Vihervaara, A.; Sergelius, C.; Vasara, J.; Blom, M. A. H.; Elsing, A. N.; Roos-Mattjus, P.; Sistonen, L. Transcriptional response to stress in the dynamic chromatin environment of cycling and mitotic cells. *Proc. Natl. Acad. Sci. USA* **2013**, *110*, E3388–97.

112. Vilaboa, N.; Boré, A.; Martin-Saavedra, F.; Bayford, M.; Winfield, N.; Firth-Clark, S.; Kirton, S. B.; Voellmy, R. New inhibitor targeting human transcription factor HSF1: effects on the heat shock response and tumor cell survival. *Nucleic Acids Res.* **2017**, *45*, 5797–5817.

113. Le Masson, F.; Razak, Z.; Kaigo, M.; Audouard, C.; Charry, C.; Cooke, H.; Westwood, J. T.; Christians, E. S. Identification of heat shock factor 1 molecular and cellular targets during embryonic and adult female meiosis. *Mol. Cell. Biol.* **2011**, *31*, 3410–3423.

114. Korfanty, J.; Stokowy, T.; Widlak, P.; Gogler-Piglowska, A.; Handschuh, L.; Podkowiński, J.; Vydra, N.; Naumowicz, A.; Toma-Jonik, A.; Widlak, W. Crosstalk between HSF1 and HSF2 during the heat shock response in mouse testes. *Int. J. Biochem. Cell Biol.* **2014**, *57*, 76–83.

115. Kus-Liśkiewicz, M.; Polańska, J.; Korfanty, J.; Olbryt, M.; Vydra, N.; Toma, A.; Widłak, W. Impact of heat shock transcription factor 1 on global gene expression profiles in cells which induce either cytoprotective or pro-apoptotic response following hyperthermia. *BMC Genomics* **2013**, *14*, 456.

116. Qu, Z.; Titus, A. S. C. L. S.; Xuan, Z.; D'Mello, S. R. Neuroprotection by Heat Shock Factor-1 (HSF1) and Trimerization-Deficient Mutant Identifies Novel Alterations in Gene Expression. *Sci. Rep.* **2018**, *8*, 17255.

117. Hahn, J.-S.; Hu, Z.; Thiele, D. J.; Iyer, V. R. Genome-wide analysis of the biology of stress responses through heat shock transcription factor. *Mol. Cell. Biol.* **2004**, *24*, 5249–5256.
